# Incidence and etiology of infectious diarrhea from a facility-based surveillance system in Guatemala, 2008–2012

**DOI:** 10.1186/s12889-019-7720-2

**Published:** 2019-10-22

**Authors:** Wences Arvelo, Aron J. Hall, Olga Henao, Beatriz Lopez, Chris Bernart, Juan C. Moir, Lissette Reyes, Susan P. Montgomery, Oliver Morgan, Alejandra Estevez, Michele B. Parsons, Maria R. Lopez, Gerry Gomez, Jan Vinje, Nicole Gregoricus, Umesh Parashar, Eric D. Mintz, John McCracken, Joe P. Bryan, Kim A. Lindblade

**Affiliations:** 1 Center for Global Health, US Centers for Disease Control and Prevention, 1600 Clifton Road, NE, Mailstop V24-5, Atlanta, GA 30333 USA; 2 National Center for Immunization and Respiratory Diseases, CDC, Atlanta, USA; 3 National Center for Emerging and Zoonotic Infectious Diseases, CDC, Atlanta, USA; 4 Centro de Estudios en Salud, Universidad del Valle de Guatemala, Guatemala City, Guatemala; 5 Ministry of Public Health and Welfare, Guatemala City, Guatemala

## Abstract

**Background:**

Diarrhea is a major cause of morbidity and mortality, yet incidence and etiology data are limited. We conducted laboratory-based diarrhea surveillance in Guatemala.

**Methods:**

A diarrhea case was defined as ≥3 loose stools in a 24-h period in a person presenting to the surveillance facilities. Epidemiologic data and stool specimens were collected. Specimens were tested for bacterial, parasitic, and viral pathogens. Yearly incidence was adjusted for healthcare seeking behaviors determined from a household survey conducted in the surveillance catchment area.

**Results:**

From November 2008 to December 2012, the surveillance system captured 5331 diarrhea cases; among these 1381 (26%) had specimens tested for all enteric pathogens of interest. The adjusted incidence averaged 659 diarrhea cases per 10,000 persons per year, and was highest among children aged < 5 years, averaging 1584 cases per 10,000 children per year. Among 1381 (26%) specimens tested for all the pathogens of interest, 235 (17%) had a viral etiology, 275 (20%) had a bacterial, 50 (4%) had parasites, and 86 (6%) had co-infections. Among 827 (60%) specimens from children aged < 5 years, a virus was identified in 196 (23%) patients; 165 (20%) had norovirus and 99 (12%) rotavirus, including co-infections. Among 554 patients aged ≥5 years, 103 (19%) had a bacterial etiology, including diarrheagenic *Escherichia coli* in 94 (17%) cases, Shigella spp. in 31 (6%), Campylobacter spp. in 5 (1%), and Salmonella spp. in 4 (1%) cases. Detection of Giardia and Cryptosporidium was infrequent (73 cases; 5%).

**Conclusions:**

There was a substantial burden of viral and bacterial diarrheal diseases in Guatemala, highlighting the importance of strengthening laboratory capacity for rapid detection and control and for evaluation of public health interventions.

## Background

Diarrheal diseases are a major cause of morbidity and mortality worldwide, particularly among children and the elderly [1–3]. In developing countries, diarrhea is the second leading cause of mortality in children < 5 years of age [4]. Annually, approximately four billion diarrhea episodes and 1.3 million diarrhea-related deaths occur worldwide [1]. Despite the magnitude of diarrheal diseases, these are only estimates derived from different data sources gathered through varying methodologies [1, 4–6]. These estimates have limitations that may lead to underreporting and an underestimation of the actual burden. For example, healthcare workers might face challenges for consistently reporting diarrheal episodes. Milder illnesses often never reach a reporting facility. Lastly, healthcare utilization practices are complex, and social, economic, geographic, and individual factors affect healthcare seeking behaviors [7, 8].

The US Centers for Disease Control and Prevention (CDC) in collaboration with the *Ministerio de Salud Publico y Asistencia Social* and the *Universidad del Valle de Guatemala* (UVG), initiated an active laboratory-based surveillance system for diarrheal, respiratory, febrile illness, and acute infectious neurological diseases in two departments in Guatemala. Coupled with laboratory testing, the main objectives of this surveillance system were to determine the etiology-specific burden of these syndromes and serve as a platform for evaluating the effectiveness or impact of interventions such as, vaccines, zinc, and campaigns for use of oral rehydration therapy. Given the limited information on healthcare utilization practices in Guatemala, before launching the surveillance system we also conducted household surveys to understand healthcare utilization practices and adjust disease incidence. In this report we describe healthcare utilization practices for diarrhea in two sites in Guatemala, estimate the incidence of diarrheal disease, and describe the predominant bacterial, viral, and parasitic pathogens detected. More comprehensive pathogen specific clinical characterization and burden of illness has been published elsewhere.

## Methods

### Sites

**Fig. 1:**
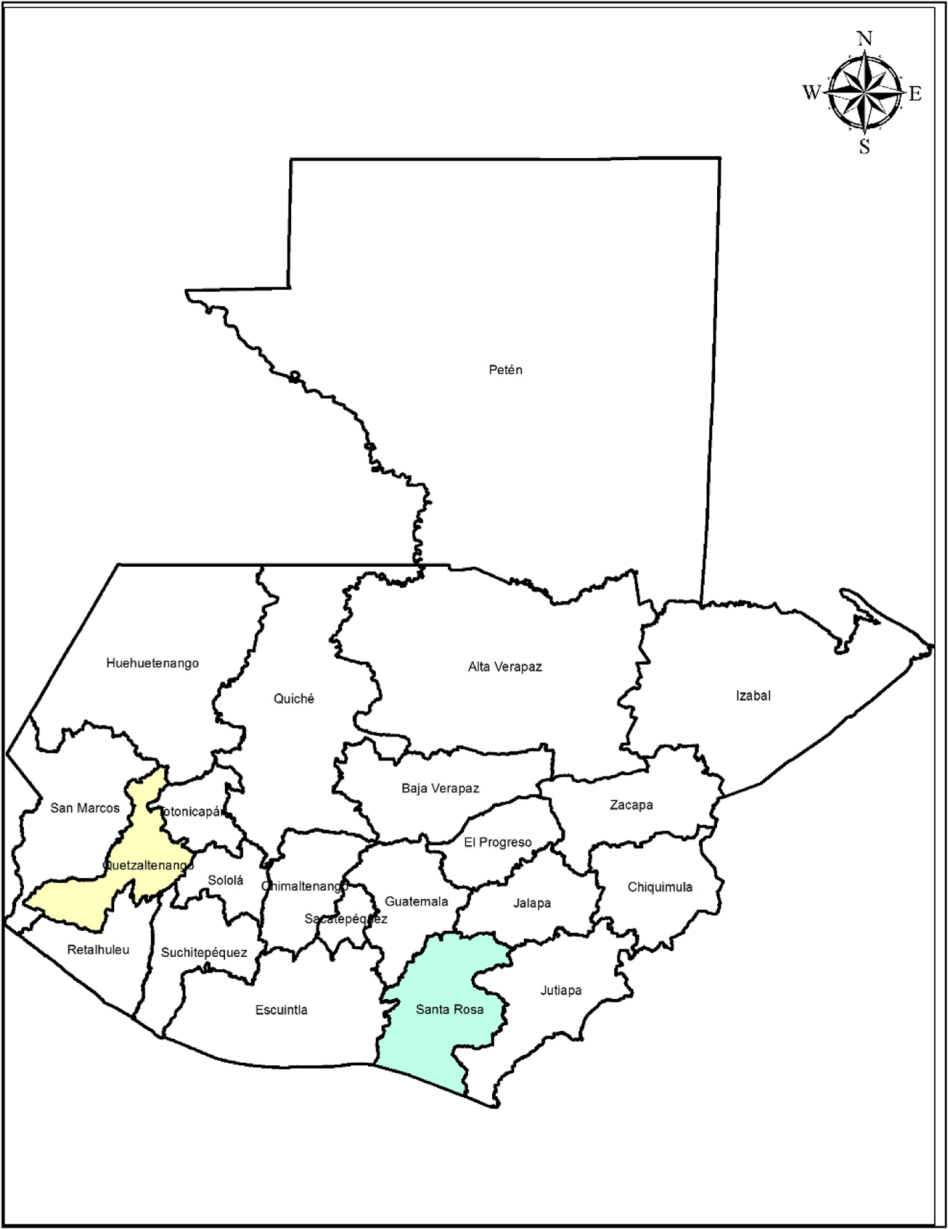
Map of Guatemala highlighting Santa Rosa and Quetzaltenango, the two departments where the healthcare seeking practices surveys and the surveillance system for diarrheal diseases were implemented

Guatemala is divided into 22 departments which are in turn divided into municipalities. The household surveys and the surveillance system were conducted in the departments of Santa Rosa and Quetzaltenango (Fig. 1). The sites were purposely selected based on logistical and political factors. In the 2002 national census, the most recently available census at the time of the study, the department of Santa Rosa had a population of 300,928, and Quetzaltenango had a population of 623,494 persons.

Government healthcare facilities in these departments included hospitals, and ambulatory centers. Cuilapa Regional Hospital is a 176-bed referral hospital in Santa Rosa, and serves Santa Rosa and the neighboring departments of Jutiapa and Jalapa. The Hospital del Occidente (Western Regional Hospital) in Quetzaltenango is a 425-bed hospital, and also serves the neighboring departments of Huehuetenango, San Marcos, Totonicapán. Other sources of healthcare in these departments include smaller public hospitals, private clinics, private hospitals, pharmacies, and traditional healers.

Before initiating surveillance, we conducted household surveys to describe healthcare utilization practices and treatment for diarrheal episodes. In Guatemala, the National Census Bureau defines an enumeration area, as the discrete geographical area for counting individuals. In the Department of Santa Rosa, we stratified enumeration areas obtained from the Guatemala National Census Bureau by those with immediate access to a hospital or health centers and those without immediate access to these health facilities. Selection of enumeration areas was conducted by probability proportional to population size based on the 2002 census data. Thirty enumeration areas were selected per stratum for a total of 60 enumeration areas. Sketch maps denoting the general layout of main structures within each enumeration area were obtained, and 20 structures were randomly selected. Once the field teams reached a selected structure they attempted to interview all persons living in the household. A household was defined as an individual or group of individuals who lived in that residential structure, including sleeping most of the nights and eating most of their meals, for at least 6 months in the 12 months prior to interview. If a structure was determined to be abandoned or non-residential, it was replaced with the next nearest until 20 households in each enumeration area were enrolled.

In Quetzaltenango the study population included persons living in municipalities served by Western Regional Hospital, which based on hospital records included the capital city and its nine surrounding rural municipalities of Almolonga, Cantel, Concepción Chiquirichapa, La Esperanza, Olintepeque, Salcajá, San Juán Ostuncalco, San Mateo and Zunil. We selected two representative samples: one from the higher density urban area in the capital, and another from the surrounding rural and semi-urban areas. In the urban area we took a stratified simple random sample of households using geospatial sampling. The district was first stratified into 26 strata based on administrative areas. We used proportional allocation (self-weighting) to determine the number of households required in each stratum by dividing the population of each stratum by the total population of the district and multiplying by 710, the number of required households based on sample size calculations. Within each stratum we conducted a simple random selection of households without replacement using geospatial sampling, by taking a random selection of coordinates in each stratum and selecting the nearest household to that coordinate. To achieve this, we first demarcated boundaries for each urban stratum using an engineering map in AutoCAD software provided by the municipal planning department. We then plotted the boundary coordinates onto high-resolution aerial photographs available from the Guatemalan *Instituto Geográfico Nacional* (National Geographic Institute) that had been overlaid onto a topographic map. The aerial images were taken in 2006. Within each demarcated stratum we randomly selected coordinates, which we refer to as sample points. We then plotted the sample points onto the high-resolution aerial photographs. We then selected the nearest residential structure within a 100 m radius from the sample point. If two or more residential structures were a similar distance from the sample point, we used a random number table to select one of them. If there were two or more households in the selected residential structure, we used a simple random number table to select one of them. If no residential structure was within 100 m of the sample point, the sample point was replaced. For rural areas we took a two-stage cluster sample where the first stage was a sample of communities and the second stage was a sample of households within each selected community. For the first sampling stage we selected 35 clusters of 39 households to achieve the desired sample size of 1365 households. We selected clusters using the PROC SURVEYSELECT command in Statistical Analysis System version 9.3 ([SAS], SAS Institute, Cary, NC). For the second sampling stage we took a simple random sample of households within each cluster using geospatial sampling. This process was similar to the one described above for the urban area except that, for demarcation of communities, we visited each community and with a local guide demarcated the communities’ boundaries using a handheld global positioning system (GPS) device.

For both surveys, interviews were conducted in person using a standardized questionnaire, administered in the local language by trained interviewers. The survey asked about episodes of diarrhea during the last 30 days before the interview for all members of the household. Question were asked to characterize the diarrheal episode, such as number of episodes in 24 h, blood in stools, fever, etc. We also asked about healthcare seeking practices, and whether they visited a hospital, ambulatory clinic, private physician, pharmacy, traditional healer, among others. In addition, we asked about treatments during that episode of diarrhea, including antibiotics, rehydration solutions, herbal remedies, etc. The survey also collected information on demographic and socioeconomic characteristics (Additional file 1). All persons who had lived in the house for 6 months or more in the 12 months before the interview were eligible. Infants < 6 months of age were included if they lived in the household since birth. For anyone who died < 30 days before the interview, young children, or those absent at the time of the interview, questions were asked of a surrogate household member whether the deceased had experienced diarrhea in the 30 days prior to dying. In Santa Rosa, data were collected on paper forms and scanned into a Microsoft Access 2003 (Microsoft Corporation, Seattle, WA) database using TeleForms (Hewlett Packard Enterprise, Palo Alto, CA). In Quetzaltenango, the survey was conducted using personal digital assistant devices (PDAs).

### Surveillance

**Fig. 2:**
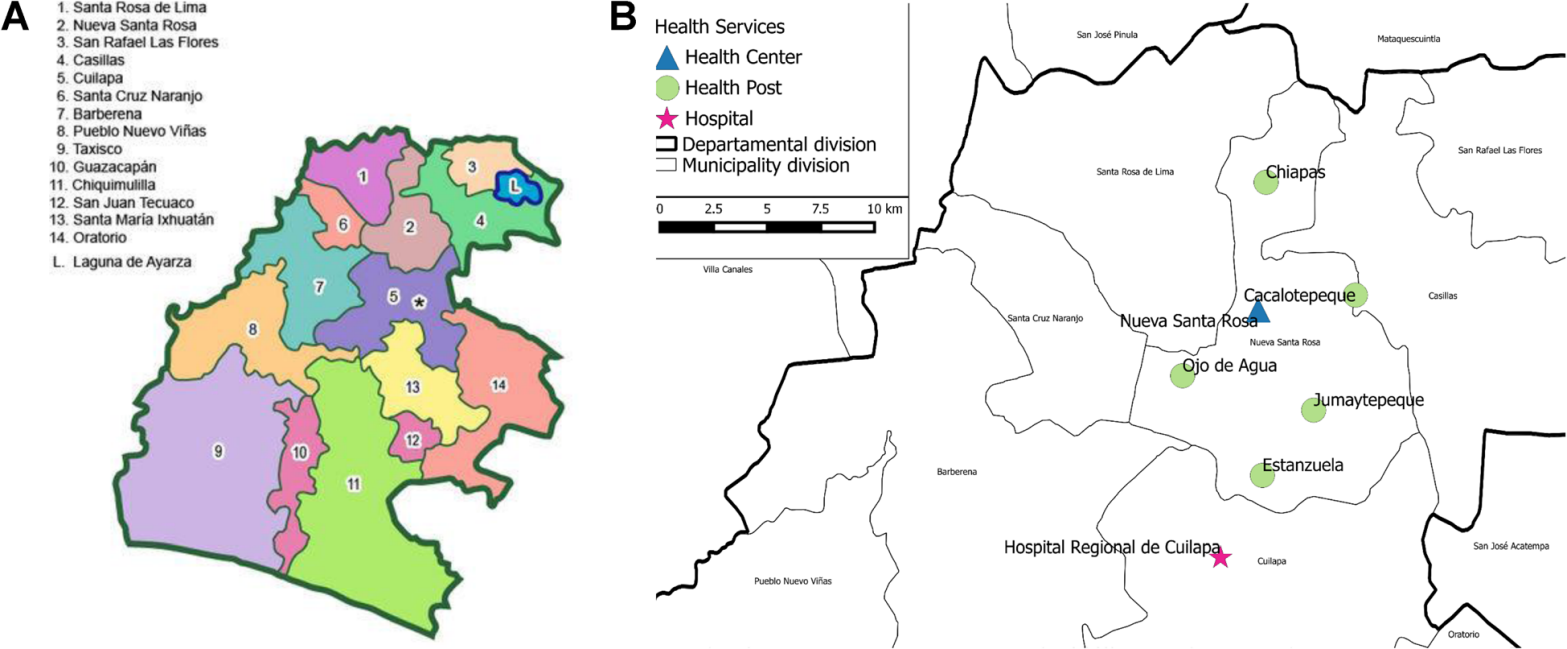
**a**: Political map showing municipalities in the Department of Santa Rosa. The Health Utilization Survey was conducted across the entire Department of Santa Rosa. The municipality of Nueva Santa Rosa is number 2 on the map at left and the capital of Cuilapa where the regional hospital is located is marked with a star. Surveillance for disease at the hospital includes all municipalities except the coastal municipalities of Taxisco, Guazacapan and Chiquimulilla as residents of these municipalities are more likely to go to a neighboring department (Esquintla) because of transportation access. **b**: Municipality of Nueva Santa Rosa where disease surveillance is conducted including the town of Nueva Santa Rosa where the health center is located and the health posts in Cacalotepeque, Chapas, Estanzuelas, Jumaytepeque, and Ojo de Agua

**Fig. 3:**
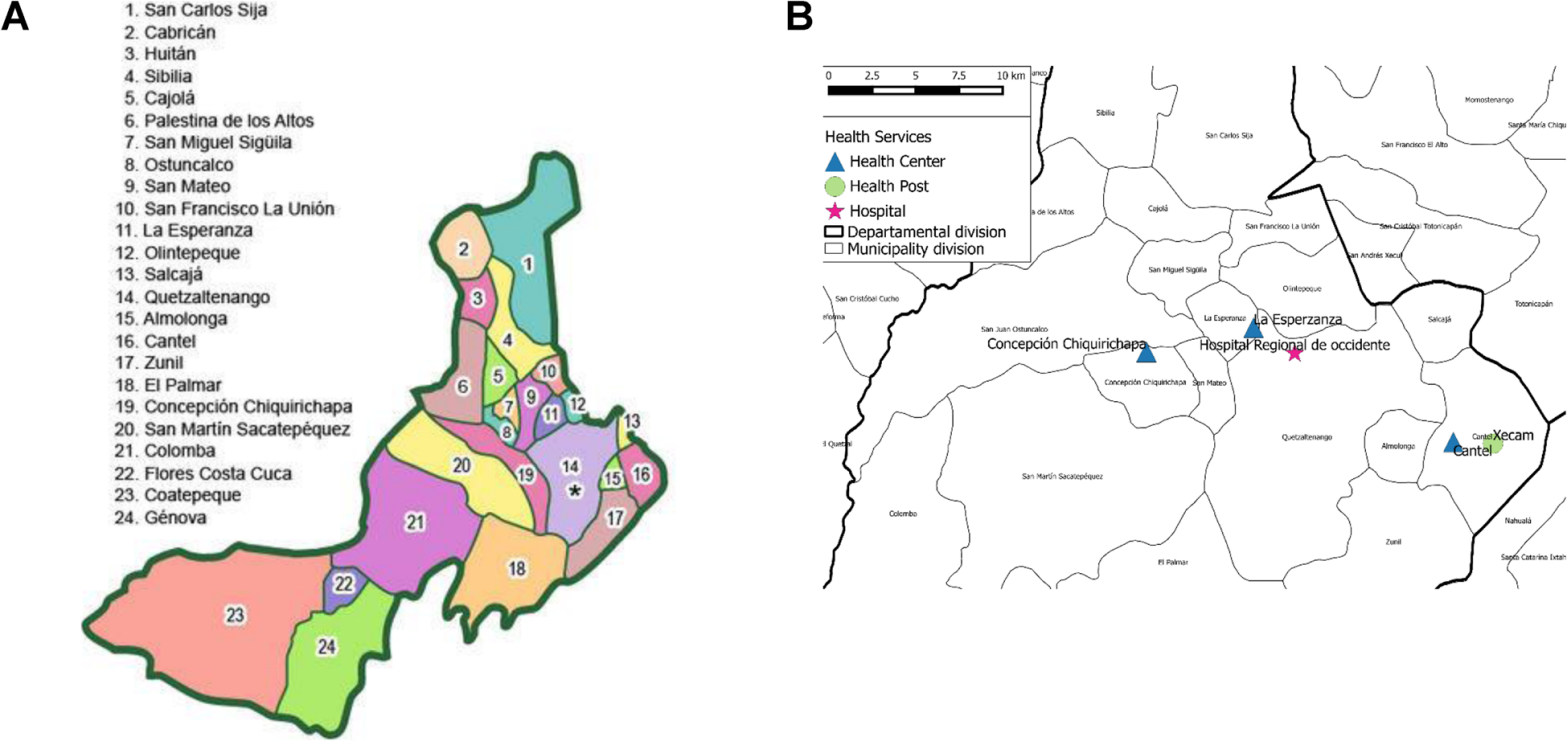
**a:** Political map showing municipalities in the Department of Quetzaltenango. Quetzaltenango, the capital and the location of the regional hospital and its nine surrounding rural municipalities of Almolonga (15), Cantel (16), Concepción Chiquirichapa (19), La Esperanza (11), Olintepeque (12), Salcajá (13), San Juán Ostuncalco (8), San Mateo (9) and Zunil (17) are the locations of the health utilization survey. Disease surveillance was conducted at the Western Regional Hospital capturing hospitalized cases from across the department of Quetzaltenango, and in four surrounding ambulatory facilities, namely the health centers in Cantel, Concepcion Chiquirichapa, La Esperanza, and Xecam. **b**: Health Centers in Concepcion Chiqurichapa, La Esperanza, Cantel and Xecam and Hospital Regional de Occidente where disease surveillance was conducted in Quetzaltenango Department

In Santa Rosa (Fig. 2a) the surveillance system initiated in July 2007 at Cuilapa Regional Hospital and the six health centers (Nueva Santa Rosa, Cacalotepeque, Chapas, Estanzuelas, Jumaytepeque, and Ojo de Agua- Fig. 2b) in the municipality of Nueva Santa Rosa. In Quetzaltenango surveillance began in February 2009 at the Western Regional Hospital and the four surrounding health centers in Cantel, Concepcion Chiquirichapa, La Esperanza, and Xecam (Fig. 3a). Surveillance systems in both sites are ongoing.

At the hospitals, trained nurses identified patients admitted with signs or symptoms suggestive of diarrhea by reviewing ward registers for diarrhea-related admission diagnoses or chief complaints emergency department registries. At ambulatory facilities, nurses screened patients presenting for treatment for diarrhea-related symptoms. A case of acute diarrhea was defined as ≥3 loose stools in a 24-h period during the previous 7 days in a person of any age admitted to the hospital or presenting to the health centers. Patients with diarrhea > 7 days were excluded. Clinical and epidemiologic data were collected using standardized questionnaires (Additional file 2).

We attempted to collect whole stool specimens from enrolled patients. When whole stool was not obtainable for children < 5 years old, we collected a rectal swab [9]. Specimens were kept at 4 °C and transported within 24 h to the laboratory. Specimens were tested for enteric viruses, bacteria, and parasites [7, 10, 11]. Rotavirus testing was done via qualitative enzyme immunoassay (EIA) for the detection of Group A rotavirus with the IDEIA Rotavirus test (Thermo Fisher Scientific Inc., Waltham, MA) [7]. Norovirus testing for genogroup I and II was done by real-time reverse transcription quantitative polymerase chain reaction (RT-qPCR) [12]. We tested for enteric bacteria including *Campylobacter*, *Salmonella*, *Shigella*, and diarrheagenic *Escherichia coli*^12^. *Salmonella* and *Shigella* were cultured using routine bacteriological methods, while for *Campylobacter* we used selective agar base, Karmali (Oxoid, Basingstoke, UK) and incubated at 42 °C for 48 h under microaerophilic conditions provided by the CampyGen™ generating system (Oxoid, Basingstoke, UK)^13^. *E. coli* testing was performed on bulk stool specimens by conventional PCR to detect the presence of specific virulence determinants of enterotoxigenic *E. coli,* enteropathogenic *E. coli and* shiga-toxin producing *E. coli.*^*14,15*^*.* Examination for protozoa and soil transmitted helminths was conducted microscopically by direct smear examination. *Cryptosporidium* spp. and Giardia were also detected by direct fluorescent antibodies (http://www.meridianbioscience.com/diagnostic-products/cryptosporidium-and-giardia/merifluor/merifluor-cryptosporidium-and-giardia.aspx)^9^.

### Data management and analysis

Data were collected in the field by trained staff using PDAs. Data were managed and stored using Microsoft SQL Server (Microsoft Corporation, Seattle, WA). All data are kept in secured servers at the UVG given they contain personally identifiable and sensitive clinical information protected by the Health Insurance Portability and Accounting Act (HIPAA). Redacted deidentified versions of the data are available upon request.

We analyzed data using SAS version 9.1 (SAS Institute, Cary, NC). From the household surveys we calculated the proportion of patients seeking health-care as the number of participants who reported diarrhea and the proportion who sought care at a government health facility by age group, with 95% confidence intervals (CI) taking into account the survey design. For incidence calculations we used the cases detected by the surveillance system, including from hospitals and ambulatory sites. Yearly incidences were calculated by dividing the total number of diarrhea cases (hospitalized and ambulatory) by the mid-year population. Adjusted rates were calculated by dividing crude rates by the proportion of patients with diarrhea who reported seeking health care at a government facility in the household surveys. Each mid-year population was estimated from the most recently available 2002 national census data, as reported by the Guatemalan National Institute for Statistics.

We also conducted an etiologic analysis limited to those diarrhea cases captured from July 2008 to July 2009 for which testing was done for all the bacterial, parasitic, and viral enteric pathogens under surveillance. We defined a bacterial etiology as a case with *Campylobacter* spp., diarrheagenic *E. coli* (including enterotoxigenic, enteropathogenic, and Shiga-toxin producing *E. coli), Salmonella* spp.*,* or *Shigella* spp.; a viral etiology as a case with confirmed rotavirus or norovirus; and a parasitic etiology as one with *Giardia lamblia* (also known as *Giardia intestinalis or Giardia duodenalis)* or *Cryptosporidium* spp. The etiologic analysis was also stratified by age group and by type of facility (hospital and ambulatory).

### Ethics approval and consent to participate

Protocols for both the household surveys and the surveillance system received approval from the institutional review boards of the US Centers for Disease Control and Prevention (Atlanta, GA; CDC IRB protocol #5150), and of the Universidad del Valle de Guatemala (Guatemala City, Guatemala). In addition, the protocol was reviewed and received approval from the Guatemalan Ministry of Public Health and Welfare (Guatemala City, Guatemala). The protocols included institutional review board approval to enroll minors < 17 years of age with consent from the parent or caregiver, and assent if the child was 7 years of age or older.

For the household surveys, heads of household > 18 years old were asked for written, informed consent for them and the members of the household to participate in the survey. Head of household was defined as the primary caregiver of most children in household or the oldest person > 18 years old in the household. All other members of the household > 18 years old were also asked to provide verbal consent, and children aged 7 through 17 years provided verbal assent, as approved by institutional review boards. Verbal consent was approved as there was no risk for the household members to participate in a small subset of question from the survey, and heads of households had provided written informed consent.

For the surveillance system, patients ≥18 years old and caregivers of children < 18 years old had to provide written, informed consent in order for them or their children to participate in the surveillance. For children aged 7 through 17 years, written assent was required in addition the caregivers written consent. For children younger than seven years we only obtained written consent to participate from their caregivers, as approved by the institutional review boards. Trained nurses informed participants about the study, including that their participation was voluntary and confidential. An informational sheet with a description of the study and contact information for the investigators was also provided. Signed consent forms were delivered to the investigators at the UVG daily.

## Results

### Household surveys

In Santa Rosa we enrolled 5449 persons in 1131 households from October 10 through December 13, 2006. Diarrhea in the last 30 days was reported by 375 [7%; 95% CI: 6–8%] persons. Among those who reported diarrhea, 184 (49%; 95% CI: 44–54%) were female and 90 (24%; 95% CI: 20–29%) were children < 5 years old. In terms of healthcare seeking practices, 228 (61%; 95% CI: 56–66%) persons sought care for their diarrheal illness outside their home, and 87 (23%; 95% CI: 19–28%) visited a government healthcare facility (Table 1). In Quetzaltenango we enrolled 9668 persons in 1851 households from March 3 through June 11, 2009. Diarrhea in the last 30 days was reported by 747 (8%; 95% CI: 7–8%) persons. Among those who reported diarrhea, 380 (51%; 95% CI: 47–54%) were female and 167 (19%; 95% CI: 15–22%) were children < 5 years old. In terms of healthcare seeking practices, 480 (65%; 95% CI: 61–69%) persons sought care for their diarrheal illness outside the home, and 360 (48%; 95% CI: 45–52%) visited a government healthcare facility (Table 1).

**Table 1:** Healthcare seeking practices among persons who reported having diarrhea during a community household survey in Santa Rosa and Quetzaltenango, Guatemala

Age group (years)	Santa Rosa			Quetzaltenango		
	Reported Diarrhea N	Sought Care at Surveillance Facility		Reported Diarrhea N	Sought Care at Surveillance Facility	
		n	(%, 95 CI)		n	(%, 95 CI)
< 1	13	6	(46, 23–71)	22	9	(41, 23–61)
1–4	77	29	(38, 28–49)	146	35	(24, 18–32)
5–19	111	21	(19, 13–27)	213	22	(10, 7–15)
20–49	86	17	(20, 13–29)	237	29	(12, 9–17)
> 50	88	14	(16, 10–25)	129	11	(9, 5–15)
Total	375	87	(23, 19–28)	747	106	(14, 12–17)

### Surveillance

**Fig. 4:**
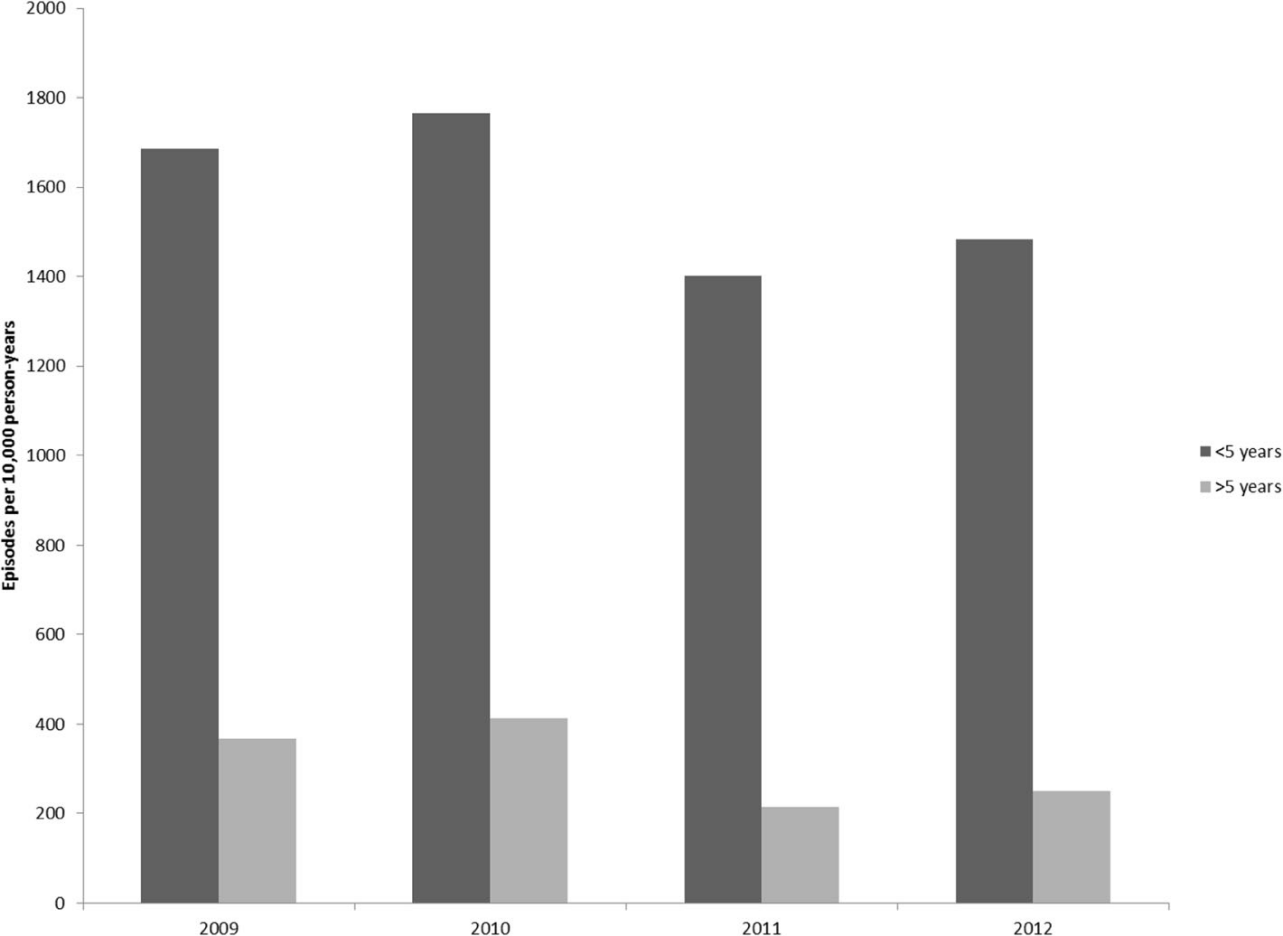
Adjusted Incidence of Diarrhea by Age Group from Surveillance in Santa Rosa and Quetzaltenango, Guatemala, 2009–2012

From November 2008 to December 2012, there were 5331 case detected by the surveillance system; 3947 cases were from patients residing in the Department of Santa Rosa and 1384 from the Department of Quetzaltenango. The incidence of diarrhea among persons attending the government health care facilities from the catchment areas was 134/10,000 patients/year. However, the average estimated community incidence across both sites adjusted for health care utilization was 659 diarrhea cases per 10,000/persons per year (range: 587–783 diarrhea cases per 10,000 persons per year). The adjusted incidence was highest among children < 5 years old, averaging 1584 cases per 10,000 children per year (range: 1401–1767 cases per 10,000 children per year) (Fig. 4).

During 2008–2009 surveillance period, there were 1381 (26%) diarrhea cases enrolled that had specimens tested for all the pathogens of interest. Overall, 235 (17%) had only viral pathogens, 275 (20%) had only bacterial pathogens, and 50 (4%) had only parasites (Table 2). Eighty-six (6%) cases had co-infections across etiological. Among 827 (60%) specimens from children < 5 years old, a viral etiology was identified in 196 (23%): norovirus was detected in 165 (20%) and rotavirus in 99 (12%) cases (Table 3). In contrast, bacterial etiologies were the most frequently identified etiology for all age groups except among infants < 1 years old (Table 2). Excluding infants, among the 1007 patients of all other age groups, 227 (23%) had a bacterial etiology. Among patients aged ≥5 years, diarrheagenic *Escherichia coli* was detected in 94 (17%) cases, *Shigella* spp. in 30 (6%), *Campylobacter* spp. in 5 (1%), and *Salmonella* spp. in 4 (1%) cases (Table 3). Identification of protozoa (*Giardia* or *Cryptosporidium*) was infrequent with 73 cases overall. Protozoa were detected predominantly in the ambulatory setting where 63 (5%) patients had *Giardia* and 9 (1%) had *Cryptosporidium*. Among those aged 5–19 years, 9% had *Giardia* or *Cryptosporidia*. Overall 6% of patients had multiple pathogens detected, including 10% in those < 1 year old.

**Table 2:** Etiology of diarrhea by age group and sex from laboratory-based disease surveillance conducted from July 2008 to July 2009, Santa Rosa and Quetzaltenango, Guatemala

Age Group (Years)	Specimens Tested	Etiologic Category*									
		Viral		Bacterial		Parasitic		Multiple		Unknown	
< 1	294	97	33%	47	16%	3	1%	29	10%	118	40%
1–4	533	99	19%	124	23%	19	4%	34	6%	257	48%
5–19	254	15	6%	48	19%	23	9%	15	6%	153	60%
20–49	202	12	6%	34	17%	3	2%	7	4%	146	72%
> 50	98	8	8%	21	21%	1	1%	1	1%	67	68%
Total	1381	235	17%	275	20%	50	4%	86	6%	741	54%

*Etiologic category assigned based on detection of pathogens within a given category (e.g., viral, bacterial, and parasitic) and in the absence of pathogens from other categories

**Table 3:** Detection of Specific Pathogens among Diarrhea cases by Healthcare Facility Type and Age Group in Santa Rosa and Quetzaltenango, Guatemala

Pathogen Detected*	Ambulatory						Hospital					
	<5 years		≥5 years		All ages		<5 years		≥5 years		All ages	
	*n* = 630 (%)		*n* = 507 (%)		*n* = 1137 (%)		*n* = 216 (%)		*n* = 48 (%)		*n* = 264 (%)	
Bacteria												
Diarrheagenic *E. coli*	128	21%	89	18%	217	19%	33	16%	5	10%	38	15%
*Shigella*	35	6%	28	6%	63	6%	6	3%	3	6%	9	4%
*Campylobacter*	30	5%	5	1%	35	3%	7	3%	0	–	7	3%
*Salmonella*	1	< 1%	3	1%	4	< 1%	1	1%	1	2%	2	1%
Virus												
Norovirus	104	17%	30	6%	134	12%	61	30%	4	8%	65	26%
Rotavirus	50	8%	19	4%	69	6%	49	24%	0	==	49	19%
Protozoa												
*Giardia*	27	4%	34	7%	61	5%	2	1%	0	–	2	1%
*Cryptosporidium*	6	1%	1	< 1%	7	1%	2	1%	0	–	2	1%

*Multiple pathogens may be detected in a given specimen; thus, some diarrhea cases are counted in more than one row

## Discussion

We report population-based incidence estimates for diarrheal diseases across two sites in Guatemala. The highest incidence adjusted for healthcare seeking behavior was among children < 5 years of age with 1584 cases per 10,000 children per year. The etiologic profile of pathogens detected were similar to findings from a multinational study where rotaviru*s*, *Cryptosporidium*, *Shigella* and pathogenic *E. coli* were significantly associated with moderate to severe diarrhea in children [13]. In our study, among children < 1 year old the main etiologies were viral. Norovirus was found in a third of hospitalized children, and 17% from ambulatory clinics. Rotavirus accounted for 23% of hospitalizations and 8% of ambulatory visits among children < 5 years. We expect lower rates of rotavirus after introduction of the vaccine in early 2010, which will increase the relative importance of norovirus as an etiology of severe diarrheal disease in children [14]. This is consistent with other reports showing high levels of norovirus I sporadic cases as well as outbreaks in Guatemala [9, 14, 15]. Among those ≥1 years old, bacterial pathogens, particularly diarrheagenic *E. coli*, played a more prominent role. In our surveillance, 18% of ambulatory patients in this age group had diarrheagenic *E. coli*, as did 10% of those hospitalized. In addition, *Shigella* was detected in 6% and *Campylobacter* was detected in 3% overall, but *Salmonella* detection was lower than expected at 1% [16]. Likewise, *Cryptosporidium* detection was lower than in the Global Enteric Multicenter Study (GEMS) [13]. Optimal testing for *E. histolytica* was not conducted, but entamoeba cysts were seldom observed.

We also found from the health utilization surveys that although the majority of patients sought care for their illness outside the home, less than 25% went to a government health facility. Even though children < 5 years old most often sought care at a government health facility, only 51% of these children sought care at these facilities. Low utilization of government-run services for diarrhea are consistent with those of another study in a similar population in Guatemala [8, 17, 18]. In Guatemala, government facilities are responsible for reporting diarrheal diseases, and national surveillance systems such as the Rotavirus Sentinel Surveillance, located is facility-based, as in many other countries [19–21]. Our findings show the national rotavirus sentinel surveillance in Guatemala may be capturing about half of cases of diarrhea among children < 5 years of age. While these surveys conducted in two of Guatemala’s 22 administrative areas may not be representative, attempts should be made to correct local and national surveillance data intended for describing incidence and hospitalization rate estimates adjusting for healthcare seeking practices.

Our study is subject to several limitations. First, we did not include control groups for comparison of etiologic detection rates. Results from GEMS showed a high pathogen detection, particularly norovirus, *Campylobacter*, and *Giardia* in asymptomatic control children, suggesting that our data may have over-represented their importance as pathogens [13]. Second, we found much lower incidences than in other reports. A recent systematic review reported a median global incidence for diarrheal in children < 5 years of age at 3.4 episodes per person-year. The incidence in Guatemala in this systematic review was 4.3 per child year [1]. Another systematic review among older children and adults also reported a higher incidence rate than ours, but lacked data from Central and South America [4, 22]. There are several reasons why the incidence for diarrhea in these systematic reviews was higher than in our study. We used healthcare facility data and extrapolated our findings based on healthcare seeking data from household surveys, and might have overestimated healthcare seeking rates, which would lead to a lower extrapolated incidence rate. During the household surveys, we ascertained healthcare seeking practices for the 30 days prior to interview, and recall bias may have led to an underestimation. The household survey in Santa Rosa was conducted during the rotavirus season, which could have also led to an overestimation of healthcare seeking practices, resulting in lower adjusted incidence rates. Finally, we may have under-ascertained diarrhea cases presenting at surveillance facilities. In our study patients presenting to the emergency department, but not admitted to the hospital, were not eligible for inclusion. Only patients who were hospitalized were enrolled in the surveillance system, as one of the main objectives for the implementation of the surveillance system was to capture the more severe diarrhea cases. Since many patients with diarrhea receive hydration in the emergency department and are then discharged home, this may have led to much lower numbers reported as compared to other studies. Despite these limitations, this surveillance system yielded high quality results with laboratory-based detection of enteric pathogens, through consistent surveillance with dedicated nurses and laboratorians, and the use of PDAs and electronic transfer of data.

## Conclusions

We estimated that in our surveillance sites in Guatemala, approximately 1 in 15 persons suffers and seeks medical care for a diarrheal disease episode each year. Our estimates were based from active laboratory-based diarrhea surveillance among ambulatory and hospitalized patients, and observed rates were adjusted with data from surveys describing healthcare seeking practices. Pathogen specific data highlighted the importance of viruses and diarrheagenic *E. coli* as an important etiology for diarrhea in this region. *Shigella* remains an important pathogen with considerations for increase antimicrobial resistance. This investigation highlights the importance of strengthening laboratory capacity in Guatemala for rapid detection and control of health threats, and the evaluation of public health interventions to inform adequate control and prevention strategies that could reduce the burden of diarrheal diseases.

### Disclaimer

Reported findings and conclusions are those of the authors and do not necessarily represent the official position of the CDC.

## Supplementary information

**Additional file 1.** Household Survey. Questionnaire used for the household survey.

**Additional file 2.** Surveillance Questions. Questions used for patients enrolled in the surveillance system.

## Data Availability

Regarding data availability, all data are kept in secured servers at the UVG given they contain personally identifiable and sensitive clinical information protected by the Health Insurance Portability and Accounting Act (HIPAA). Redacted deidentified versions of the data are available upon request from the corresponding author.

## Abbreviations

CDC: US Centers for Disease Control and Prevention
EIA: Enzyme Immunoassay
GPS: Global Positioning System
PDAs: Personal Digital Assistant Devices
RT-qPCR: Reverse Transcription quantitative polymerase chain reaction
SAS: Statistical Analysis System
UVG: Universidad del Valle de Guatemala
