# Supplementary material for: Incidence and etiology of infectious diarrhea from a facility-based surveillance system in Guatemala, 2008–2012
Source: BMC Public Health. 2019 Oct 22;19:1340. doi: 10.1186/s12889-019-7720-2 (PMC6805345; doi:10.1186/s12889-019-7720-2)
Supplement: Supplementary file 1 — Additional file 1. Household Survey. Questionnaire used for the household survey. [file 12889_2019_7720_MOESM1_ESM.pdf]

CDC-CAP/UVG-CES
Programa Internacional de Enfermedades Emergentes
Proyecto de Vigilancia Comunitaria
Formulario de Encuesta Domiciliaria en Quetzaltenango
*-

Ubicación de la vivienda
Asignación

1) ID de punto geográfico

2) ¿Encontró una vivienda habitada cerca del punto según las reglas de selección?

- ☐ Sí
- ☐ No

3) Si NO encontró una vivienda habitada

3.1) ¿Por qué no encontró una vivienda habitada?

- ☐ No había una vivienda cerca
- ☐ No había accesso a la vivienda
- ☐ Otra

3.2) Si la razón es "Otra"

3.2.1) Especifique la razón

4) Si encontró una vivienda habitada

4.1) GPSLatitude

4.2) GPSDecimalLatitude

4.3) GPSLongitude

4.4) GPSDecimalLongitude

4.5) GPSPDOP

4.6) GPSHDOP

4.7) GPSSatallites

4.8) GPS

Registro de Visita
Visita 1

1) Fecha y hora de visita #1

[ ]

2) ¿Hay un adulto en la casa que cualifique para la entrevista?

- Sí
- No

3) Si hubo un adulto en la casa

3.1) ¿El adulto pudo responder?

- Sí
- No

3.2) Si el adulto NO pudo hacer la entrevista

3.2.1) Si no, ¿por qué?

- No tuvo la capacidad dar informacion
- Barrera de idioma
- Rechazo la encuesta
- No es conveniente

3.2.2) Si la razon es "Barrera de idioma"

3.2.2.1) ¿Qué idioma?

- Español
- K'iche'
- Mam
- Otro
- No quiere contestar

3.2.2.2) Si el idioma es "Otra"

3.2.2.2.1) Especifique

[ ]

3.3) Si el adulto pudo hacer la entrevista

3.3.1) ¿Al menos una persona ha vivido aquí por lo menos 6 meses en el último año?

- Sí
- No

3.3.2) Si en el último año por lo menos una persona ha vivido aquí por lo menos 6 meses

3.3.2.1) ¿El adulto dio consentimiento verbal?

- Sí
- No

Visita 2

1) Fecha y hora de visita #2

2) ¿Hay un adulto en la casa que cualifique para la entrevista?

- Sí
- No

3) Si hubo un adulto en la casa

3.1) ¿El adulto pudo responder?

- Sí
- No

3.2) Si el adulto NO pudo hacer la entrevista

3.2.1) Si no, ¿por qué?

- No tuvo la capacidad dar informacion
- Barrera de idioma
- Rechazo la encuesta
- No es conveniente

3.2.2) Si la razon es "Barrera de idioma"

3.2.2.1) ¿Qué idioma?

- Español
- K'iche'
- Mam
- Otro
- No quiere contestar

3.2.2.2) Si el idioma es "Otra"

3.2.2.2.1) Especifique

3.3) Si el adulto pudo hacer la entrevista

3.3.1) ¿Al menos una persona ha vivido aquí por lo menos 6 meses en el último año?

- Sí
- No

3.3.2) Si en el último año por lo menos una persona ha vivido aquí por lo menos 6 meses

3.3.2.1) ¿El adulto dio consentimiento verbal?

- Sí
- No

Visita 3

1) Fecha y hora de visita #3

2) ¿Hay un adulto en la casa que cualifique para la entrevista?

- Sí
- No

3) Si hubo un adulto en la casa

3.1) ¿El adulto pudo responder?

- Sí
- No

3.2) Si el adulto NO pudo hacer la entrevista

3.2.1) Si no, ¿por qué?

- No tuvo la capacidad dar informacion
- Barrera de idioma
- Rechazo la encuesta
- No es conveniente

3.2.2) Si la razon es "Barrera de idioma"

3.2.2.1) ¿Qué idioma?

- Español

- K'iche'
- Mam
- Otro
- No quiere contestar

3.2.2.2) Si el idioma es "Otra"

3.2.2.2.1) Especifique

3.3) Si el adulto pudo hacer la entrevista

3.3.1) ¿Al menos una persona ha vivido aquí por lo menos 6 meses en el último año?

- Sí
- No

3.3.2) Si en el último año por lo menos una persona ha vivido aquí por lo menos 6 meses

3.3.2.1) ¿El adulto dio consentimiento verbal?

- Sí
- No

Vivienda
Entrevista

1) ¿Usted es jefe/jefa de la casa?

- Hombre jefe del hogar
- Mujer jefa del hogar
- Esposa del jefe del hogar
- Hijo/a del jefe del hogar
- Otro familiar
- Otra persona (no familiar)

2) ¿Cuál es su edad? (Si NS=999; si no quiere contestar=777).

3) ¿Cuál es el último año de estudios que usted ganó?

- No asistió a la escuela
- Primaria incompleta
- Primaria completa
- Secundaria incompleta
- Secundaria completa
- Diversificado
- Superior / universitaria
- No quiere contestar

4) ¿Cómo se considera usted - indígena o no indígena?

- Indígena
- No indígena
- Otro

5) Si indígena

5.1) ¿Qué grupo indígena?

- K'iche'
- Mam
- Otro

6) ¿Que idioma hablan habitualmente los miembros de su hogar o la mayoría de ellos?

- Español
- K'iche'
- Mam
- Otro
- No quiere contestar

7) Tipo de vivienda

- Casa
- Casa de vecindad
- Apartamento

8) ¿Cuántos hogares hay en la vivienda?

9) ¿En total cuántas personas viven en su hogar? (incluya bebes y personas que hayan fallecido en el último año)

10) ¿Alguien en su hogar fuma cigarrillos?

- Sí
- No
- No quiere contestar
- No sabe

11) Si alguien en la casa fuma

11.1) ¿ Cuántas personas que viven en su hogar fuman cigarrillos? (Si NS=99, si no quiere contestar=94)

Habitantes

Inscripción

1) PersonaID

2) neumonia

3) ETI

4) diarrea

5) ¿Cuál es su nombre?

6) ¿Cuál es su apellido?

7) ¿Cuál es su edad en años? (Si es menor de un año, anote 0)

8) Si edad en años igual 0

8.1) ¿Cuál es su edad en meses? Si es menor de 1 mes, ponga 0.

9) Sexo

- Masculino
- Femenino

Tamizaje

1) ¿Esta persona fue entrevistada personalmente?

- Sí
- No

2) Si tiene 6 ó más meses

2.1) ¿En el último año ha vivido aquí por lo menos 6 meses?

- ☐ Sí
- ☐ No

3) Si meses en el hogar en el último año es 6 o más O es un bebe con menos que 6 meses

3.1) ¿Esta persona está viva?

- ☐ Sí
- ☐ No

3.2) Si esta persona está viva

3.2.1) ¿Ha tenido diarrea o asientos en el último mes?

- ☐ Sí
- ☐ No
- ☐ No Sabe

3.2.2) ¿Ha tenido fiebre o calentura en el último mes?

- ☐ Sí
- ☐ No
- ☐ No Sabe

3.2.3) ¿Ha tenido tos o dolor de garganta en el último mes?

- ☐ Sí
- ☐ No
- ☐ No Sabe

3.2.4) ¿Ha tenido dificultad para respirar o le cuesta respirar en el último año?

- ☐ Sí
- ☐ No
- ☐ No Sabe

3.2.5) ¿Ha tenido neumonía en el último año?

- ☐ Sí
- ☐ No
- ☐ No Sabe

Mortalidad

1) ¿Murió en un establecimiento médico?

- Sí
- No
- No quiere contestar
- No sabe

2) Si murió en un establecimiento médico

2.1) ¿En qué establecimiento?

- Hospital público
- Hospital privado
- Centro de salud
- Clinica privada
- Otro
- No quiere contestar
- No sabe

3) Si no murió en un establecimiento médico

3.1) ¿Se llevaron los restos a un establecimiento médico después de su muerte?

- Sí
- No
- No quiere contestar
- No sabe

3.2) Si se llevaron los restos de a un establecimiento médico después de su muerte

3.2.1) ¿En qué establecimiento?

- Hospital público
- Hospital privado
- Centro de salud
- Clinica privada
- Otro
- No quiere contestar
- No sabe

4) ¿En qué año falleció? (si NS = 99, si no quiere contestar = 94)

5) Si se murió en 2008 o 2009

5.1) ¿En qué mes falleció?

[enero ▾] (LegalValues Table Mes)

5.2) ¿Tuvo diarrea en el mes anterior a su muerte?

- ☐ Sí
- ☐ No
- ☐ No quiere contestar
- ☐ No sabe

5.3) ¿Tuvo fiebre o calentura en el mes anterior a su muerte?

- ☐ Sí
- ☐ No
- ☐ No quiere contestar
- ☐ No sabe

5.4) ¿Tuvo tos o dolor de garganta en el mes anterior a su muerte?

- ☐ Sí
- ☐ No
- ☐ No quiere contestar
- ☐ No sabe

5.5) ¿En el último año, tuvo dificultad para respirar o le cuesta respirar?

- ☐ Sí
- ☐ No
- ☐ No quiere contestar
- ☐ No sabe

5.6) ¿En el último año, tuvo neumonía?

- ☐ Sí
- ☐ No
- ☐ No quiere contestar
- ☐ No sabe

Neumonía

1) ¿Durante este enfermedad, tenía tos?

- ☐ Sí
- ☐ No

- No quiere contestar
- No sabe

2) Si tenía tos

2.1) ¿Cuántos días tenía tos? (Si le da un rango, tome el promedio y redondéele; si NS=99, si no quiere contestar = 94)

3) ¿Durante esta enfermedad, tenía dificultad para respirar o le cuesta respirar (respiración agitada)?

- Sí
- No
- No quiere contestar
- No sabe

4) Si tenía dificultad para respirar / le cuesta respirar

4.1) ¿Cuántos días duró la dificultad para respirar / le cuesta respirar? (Si le da un rango, tome el promedio y redondéelo; si NS=99, si no quiere contestar = 94)

5) ¿Durante este año, le diagnosticó un trabajador de salud o medico que tenía neumonía?

- Sí
- No
- No quiere contestar
- No sabe

6) Si durante este año, le diagnosticó un trabajador de salud que tenía neumonía

6.1) ¿Dondé le diagnosticó con la neumonía?

- Centro de salud
- Hospital público
- Clínica privada
- Hospital privado
- Otro
- No quiere contestar
- No sabe

6.2) Si otro

6.2.1) **Especifique**

7) Si es un caso de Neumonía

7.1) ¿En qué año se enfermó? (si NS = 99, si no quiere contestar = 94)

7.2) Si se enfermó en 2008 ó 2009

7.2.1) ¿En qué mes se enfermó?

enero (LegalValues Table Mes)

7.3) Durante esta enfermedad, presentó: Fiebre o calentura?

- Sí
- No
- No quiere contestar
- No sabe

7.4) Durante esta enfermedad, presentó: Chillido en el pecho

- Sí
- No
- No quiere contestar
- No sabe

7.5) Durante esta enfermedad, presentó: Perdida de conocimiento

- Sí
- No
- No quiere contestar
- No sabe

7.6) Adultos y niños ≥ 3 años

7.6.1) Durante esta enfermedad, presentó: Tos con sangre

- Sí
- No
- No quiere contestar
- No sabe

7.6.2) Durante esta enfermedad, presentó: Dolor de pecho al respirar o toser

- ☐ Sí
- ☐ No
- ☐ No quiere contestar
- ☐ No sabe

7.6.3) Durante esta enfermedad, presentó: Fatiga

- ☐ Sí
- ☐ No
- ☐ No quiere contestar
- ☐ No sabe

7.6.4) Durante esta enfermedad, presentó: Pérdida de peso

- ☐ Sí
- ☐ No
- ☐ No quiere contestar
- ☐ No sabe

7.6.5) Durante esta enfermedad, presentó: Confusión

- ☐ Sí
- ☐ No
- ☐ No quiere contestar
- ☐ No sabe

7.7) Niños menores de 3 años

7.7.1) Durante esta enfermedad, presentó: Hervor o ruido de pecho

- ☐ Sí
- ☐ No
- ☐ No quiere contestar
- ☐ No sabe

7.7.2) Durante esta enfermedad, presentó: Labios y/o uñas morados

- ☐ Sí
- ☐ No
- ☐ No quiere contestar

- [ ] No sabe

7.7.3) Durante esta enfermedad, presentó: Incapaz de mamar o beber

- [ ] Sí
- [ ] No
- [ ] No quiere contestar
- [ ] No sabe

7.7.4) Durante esta enfermedad, presentó: Tuvo que parar de mamar o beber para respirar

- [ ] Sí
- [ ] No
- [ ] No quiere contestar
- [ ] No sabe

7.7.5) Durante esta enfermedad, presentó: Disminución de actividad

- [ ] Sí
- [ ] No
- [ ] No quiere contestar
- [ ] No sabe

7.7.6) Durante esta enfermedad, presentó: Vomita todo

- [ ] Sí
- [ ] No
- [ ] No quiere contestar
- [ ] No sabe

7.7.7) Durante esta enfermedad, presentó: Convulsiones (ataques)

- [ ] Sí
- [ ] No
- [ ] No quiere contestar
- [ ] No sabe

7.8) ¿Estas molestias se desarrollaron súbitamente en un periodo de un día (24 horas) o fueron desarrollándose despacio en el transcurso de dos o tres días?

- [ ] Súbitamente
- [ ] Despacio

- [ ] No quiere contestar
- [ ] No Sabe

7.9) ¿Cuántos días en total estuvo enfermo(a)? (Si le da un rango, tome el promedio y redondéelo; si NS=99, si no quiere contestar=94)

[ ]

7.10) ¿Buscó algún tipo de ayuda fuera de su casa para curar esta enfermedad?

- [ ] Sí
- [ ] No
- [ ] No quiere contestar
- [ ] No sabe

7.11) Si NO buscó algún tipo de ayuda fuera de la casa

7.11.1) ¿Cuál era la razón principal porqué no buscó atención médica fuera de la casa? (No lea las opciones; marque sólo una).

- [ ] No creyó estar muy enfermo(a)
- [ ] Se mejoró
- [ ] Hospital estaba muy lejos
- [ ] Hospital estaba cerrado
- [ ] Falta de tiempo
- [ ] Falta de transporte
- [ ] Falta de recursos por el pasaje
- [ ] Falta de recursos por tratamiento
- [ ] Malos servicios de salud
- [ ] Barrera del idioma
- [ ] Demasiada gente en el hospital
- [ ] No podia dejar a los niños
- [ ] Se automedicó
- [ ] El esposo no permite/machismo
- [ ] La religion
- [ ] Otra
- [ ] No quiere contestar
- [ ] No sabe

7.11.2) Si otra atención medica

7.11.2.1) Especifique

7.12) Si buscó algún tipo de ayuda fuera de la casa

7.12.1) ¿A dónde fue a buscar ayuda? (Marque todas que aplican)

- ☐ Puesto de Salud
- ☐ Centro de Salud
- ☐ Hospital público
- ☐ Hospital privado
- ☐ Clínica privada
- ☐ Centro de convergencia
- ☐ Trabajador de salud
- ☐ Jornada médica
- ☐ Farmacia
- ☐ Tienda
- ☐ Medicina tradicional/natural
- ☐ Miembros de la familia
- ☐ Otro
- ☐ No quiere contestar
- ☐ No sabe

7.12.2) Si Otra ayuda

7.12.2.1) Especifique

7.12.3) ¿A dónde fue primero?

- ○ Puesto de Salud
- ○ Centro de Salud

- Hospital público
- Hospital privado
- Clínica privada
- Centro de convergencia
- Trabajador de salud
- Jornada médica
- Farmacia
- Tienda
- Medicina tradicional/natural
- Miembros de la familia
- Otro
- No quiere contestar
- No sabe

7.12.4) Si mencionó Hospital por lo menos una vez

7.12.4.1) ¿Admitieron al hospital por esta enfermedad?

- Sí
- No
- No quiere contestar
- No sabe

7.12.4.2) SI admitieron para que pasara la noche en el hospital

7.12.4.2.1) ¿Cuántas noches pasó en el hospital? (si NS=99, si no quiere contestar=94)

7.12.5) Si NO fue Hospital

7.12.5.1) ¿Personal médico le recomendó ir al hospital?

- Sí
- No
- No quiere contestar
- No sabe

7.12.5.2) Si personal médico le recomendó ir al hospital

7.12.5.2.1) ¿Cuál era la razón principal porque no llevaron al hospital? (No lea las opciones; marque sólo una).

- No creyó estar muy enfermo(a)
- Se mejoró
- Hospital estaba muy lejos
- Hospital estaba cerrado
- Falta de tiempo
- Falta de transporte
- Falta de recursos por el pasaje
- Falta de recursos por tratamiento
- Malos servicios de salud
- Barrera del idioma
- Demasiada gente en el hospital
- No podia dejar a los niños
- Se automedicó
- El esposo no permite/machismo
- La religion
- Otra
- No quiere contestar
- No sabe

7.12.5.2.2) Si Otra razon

7.12.5.2.2.1) Especifique

7.12.6) ¿Le hicieron radiografía de pecho durante su enfermedad respiratoria?

- Sí
- No
- No quiere contestar
- No sabe

7.12.7) Si le hicieron radiografía de pecho durante su enfermedad respiratoria

7.12.7.1) ¿Dónde fue tomada la radiografía de pecho?

- ☐ Hospital
- ☐ Clínica privada
- ☐ Otro
- ☐ No quiere contestar
- ☐ No sabe

7.12.7.2) Si Otro radiografia

7.12.7.2.1) Especifique

7.12.8) Si NO le hicieron radiografía de pecho durante su enfermedad respiratoria

7.12.8.1) ¿Porqué no le hicieron radiografía de pecho? (No lea las opciones; marque sólo una).

- ☐ No me dijeron que tomara una placa de pecho
- ☐ No entendí porque era necesaria
- ☐ Tenía miedo de máquinas radiográficas
- ☐ No creyó estar muy enfermo / se mejoró
- ☐ La clínica está muy lejos de la casa
- ☐ La clínica no tenía el equipo / la máquina
- ☐ No tenía tiempo para ir
- ☐ No había transporte
- ☐ El pasaje era muy caro
- ☐ El tratamiento era muy caro
- ☐ No podía dejar a los niños solos en casa
- ☐ Otra
- ☐ No quiere contestar
- ☐ No sabe

7.12.8.2) Si Otra

7.12.8.2.1) Especifique

7.13) ¿Tomó medicamento para tratar esta enfermedad?

- Sí
- No
- No quiere contestar
- No sabe

7.14) Si tomó medicamento para tratar esta enfermedad

7.14.1) ¿Tomó antibióticos para tratar esta enfermedad?

- Sí
- No
- No quiere contestar
- No sabe

ETI

1) ¿Tenía fiebre o calentura en el último mes?

- Sí
- No
- No quiere contestar
- No sabe

2) ¿Tenía tos en el último mes?

- Sí
- No
- No quiere contestar
- No sabe

3) ¿Tenía dolor de garganta en el último mes?

- Sí
- No
- No quiere contestar
- No sabe

4) Si es un caso de ETI

4.1) ¿Hace cuántos días empezó esta enfermedad? (Si NS=99, si no quiere contestar=94)

4.2) ¿Cuántos días en total estuvo/tiene de estar enfermo con esta enfermedad? (Si le da un rango, tome el promedio y redondéelo; si NS=99, si no quiere contestar = 94).

4.3) Durante esta enfermedad, presentó: Escalofríos

- Sí
- No
- No quiere contestar
- No sabe

4.4) Durante esta enfermedad, presentó: Flujo nasal

- Sí
- No
- No quiere contestar
- No sabe

4.5) Durante esta enfermedad, presentó: Dificultad para respirar o le cuesta respirar (respiración agitada)

- Sí
- No
- No quiere contestar
- No sabe

4.6) Durante esta enfermedad, presentó: Chillido en el pecho

- Sí
- No
- No quiere contestar
- No sabe

4.7) Durante esta enfermedad, presentó: Disminución en actividad / más cansado de lo normal

- Sí
- No
- No quiere contestar
- No sabe

4.8) Durante esta enfermedad, presentó: Náusea o vómitos

- Sí
- No
- No quiere contestar
- No sabe

4.9) Adultos y niños ≥ 3 años

4.9.1) Durante esta enfermedad, presentó: Dolor de cabeza

- Sí
- No
- No quiere contestar
- No sabe

4.9.2) Durante esta enfermedad, presentó: Dolor de musculos

- Sí
- No
- No quiere contestar
- No sabe

4.10) ¿Buscó algún tipo de ayuda fuera de su casa para curar esta enfermedad?

- Sí
- No
- No quiere contestar
- No sabe

4.11) Si NO buscó algún tipo de ayuda fuera de la casa

4.11.1) ¿Cuál era la razón principal porqué no buscó atención médica fuera de la casa? (No lea las opciones; marque sólo una).

- No creyó estar muy enfermo(a)
- Se mejoró
- Hospital estaba muy lejos
- Hospital estaba cerrado
- Falta de tiempo
- Falta de transporte
- Falta de recursos por el pasaje
- Falta de recursos por tratamiento

- Malos servicios de salud
- Barrera del idioma
- Demasiada gente en el hospital
- No podia dejar a los niños
- Se automedicó
- El esposo no permite/machismo
- La religion
- Otra
- No quiere contestar
- No sabe

4.11.2) Si otra razon no atencion medica

4.11.2.1) Especifique

4.12) Si buscó algún tipo de ayuda fuera de la casa

4.12.1) ¿A dónde fue a buscar ayuda? (Marque todas que aplican)

- Puesto de Salud
- Centro de Salud
- Hospital público
- Hospital privado
- Clínica privada
- Centro de convergencia
- Trabajador de salud
- Jornada médica
- Farmacia
- Tienda
- Medicina tradicional/natural
- Miembros de la familia
- Otro

- [ ] No quiere contestar
- [ ] No sabe

4.12.2) Si Otro

4.12.2.1) Especifique

4.12.3) ¿A dónde fue primero?

- ( ) Puesto de Salud
- ( ) Centro de Salud
- ( ) Hospital público
- ( ) Hospital privado
- ( ) Clínica privada
- ( ) Centro de convergencia
- ( ) Trabajador de salud
- ( ) Jornada médica
- ( ) Farmacia
- ( ) Tienda
- ( ) Medicina tradicional/natural
- ( ) Miembros de la familia
- ( ) Otro
- ( ) No quiere contestar
- ( ) No sabe

4.12.4) Si mencionó Hospital por lo menos una vez

4.12.4.1) ¿Admitieron al hospital?

- ( ) Sí
- ( ) No
- ( ) No quiere contestar
- ( ) No sabe

4.12.4.2) SI admitieron para que pasara la noche en el hospital

4.12.4.2.1) ¿Cuántas noches pasó en el hospital? (si NS=99, si no

quiere contestar=94)

4.12.5) Si NO fue Hospital

4.12.5.1) ¿Personal médico le recomendó ir al hospital?

- Sí
- No
- No quiere contestar
- No sabe

4.12.5.2) Si personal médico le recomendó ir al hospital

4.12.5.2.1) ¿Cuál era la razón principal porque no lo llevaron al hospital? (No lea las opciones; marque sólo una).

- No creyó estar muy enfermo(a)
- Se mejoró
- Hospital estaba muy lejos
- Hospital estaba cerrado
- Falta de tiempo
- Falta de transporte
- Falta de recursos por el pasaje
- Falta de recursos por tratamiento
- Malos servicios de salud
- Barrera del idioma
- Demasiada gente en el hospital
- No podia dejar a los niños
- Se automedicó
- El esposo no permite/machismo
- La religion
- Otra
- No quiere contestar
- No sabe

4.12.5.2.2) Si Otra razon no hospital

4.12.5.2.2.1) Especifique

4.13) ¿Tomó medicamento para tratar esta enfermedad?

- Sí
- No
- No quiere contestar
- No sabe

4.14) Si tomó medicamento para tratar esta enfermedad

4.14.1) ¿Tomó antibióticos para tratar esta enfermedad?

- Sí
- No
- No quiere contestar
- No sabe

Diarrea

1) ¿Hace cuántos días empezó el último episodio de diarrea o asientos? (Si NS=99, si no quiere contestar=94)

2) ¿Cuántos días en total le duró la diarrea / los asientos? (Si le da un rango, tome el promedio y redondéelo; si NS=99, si no quiere contestar=94)

3) ¿En su peor día completo (24 horas) con diarrea o asientos, cuántos asientos líquidos tuvo? (Si NS=99, si no quiere contestar=94)

4) Si es un caso de diarrea

4.1) ¿Durante esta diarrea o asientos tuvo alguna de las siguientes molestias:? Diarrea o asientos con sangre

- Sí
- No
- No quiere contestar
- No sabe

4.2) ¿Durante esta diarrea o asientos tuvo alguna de las siguientes

molestias: ? Vómitos

- Sí
- No
- No quiere contestar
- No sabe

4.3) Si Vomitos:

4.3.1) ¿Cuántos días de vómitos? (NS=99, No quiere contestar = 94)

4.4) ¿Durante esta diarrea o asientos tuvo alguna de las siguientes molestias: ? Fiebre, calentura o escalofríos

- Sí
- No
- No quiere contestar
- No sabe

4.5) ¿Durante esta diarrea o asientos tuvo alguna de las siguientes molestias: ? Más sed de lo normal

- Sí
- No
- No quiere contestar
- No sabe

4.6) ¿Durante esta diarrea o asientos tuvo alguna de las siguientes molestias: ? Irritabilidad

- Sí
- No
- No quiere contestar
- No sabe

4.7) ¿Durante esta diarrea o asientos tuvo alguna de las siguientes molestias: ? Ojos hundidos

- Sí
- No
- No quiere contestar
- No sabe

4.8) ¿Durante esta diarrea o asientos tuvo alguna de las siguientes

molestias: ? Disminución en actividad / más cansado de lo normal

- Sí
- No
- No quiere contestar
- No sabe

4.9) ¿Durante esta diarrea o asientos tuvo alguna de las siguientes molestias: ? Bebe menos de lo usual

- Sí
- No
- No quiere contestar
- No sabe

4.10) ¿Buscó algún tipo de ayuda fuera de su casa para curar esta diarrea?

- Sí
- No
- No quiere contestar
- No sabe

4.11) Si NO buscó algún tipo de ayuda fuera de la casa

4.11.1) ¿Cuál era la razón principal porqué no buscó atención médica fuera de la casa? (No lea las opciones; marque sólo una).

- No creyó estar muy enfermo(a)
- Se mejoró
- Hospital estaba muy lejos
- Hospital estaba cerrado
- Falta de tiempo
- Falta de transporte
- Falta de recursos por el pasaje
- Falta de recursos por tratamiento
- Malos servicios de salud
- Barrera del idioma
- Demasiada gente en el hospital
- No podia dejar a los niños

- Se automedicó
- El esposo no permite/machismo
- La religion
- Otra
- No quiere contestar
- No sabe

4.11.2) Si otra razon no busco atencion medica

4.11.2.1) Especifique

4.12) Si buscó algún tipo de ayuda fuera de la casa

4.12.1) ¿A dónde fue a buscar ayuda? (No lea las opciones; marque TODAS las que apliquen)

- [ ] Puesto de Salud
- [ ] Centro de Salud
- [ ] Hospital público
- [ ] Hospital privado
- [ ] Clínica privada
- [ ] Centro de convergencia
- [ ] Trabajador de salud
- [ ] Jornada médica
- [ ] Farmacia
- [ ] Tienda
- [ ] Medicina tradicional/natural
- [ ] Miembros de la familia
- [ ] Otro
- [ ] No quiere contestar
- [ ] No sabe

4.12.2) Si Otro lugar ayuda

4.12.2.1) Especifique

4.12.3) ¿A dónde fue primero?

- ☐ Puesto de Salud
- ☐ Centro de Salud
- ☐ Hospital público
- ☐ Hospital privado
- ☐ Clínica privada
- ☐ Centro de convergencia
- ☐ Trabajador de salud
- ☐ Jornada médica
- ☐ Farmacia
- ☐ Tienda
- ☐ Medicina tradicional/natural
- ☐ Miembros de la familia
- ☐ Otro
- ☐ No quiere contestar
- ☐ No sabe

4.12.4) Si mencionó Hospital por lo menos una vez

4.12.4.1) ¿Admitieron al hospital?

- ☐ Sí
- ☐ No
- ☐ No quiere contestar
- ☐ No sabe

4.12.4.2) Si admitieron para que pasara la noche en el hospital

4.12.4.2.1) ¿Le administraron suero inyectado o suero en la vena como parte del tratamiento para la diarrea o los asientos?

- ☐ Sí
- ☐ No

- No quiere contestar
- No sabe

4.12.5) Si NO fue Hospital

4.12.5.1) ¿Personal médico le recomendó ir al hospital?

- Sí
- No
- No quiere contestar
- No sabe

4.12.5.2) Si personal médico le recomendó ir al hospital

4.12.5.2.1) ¿Cuál era la razón principal porque no lo llevaron al hospital? (No lea las opciones; marque sólo una).

- No creyó estar muy enfermo(a)
- Se mejoró
- Hospital estaba muy lejos
- Hospital estaba cerrado
- Falta de tiempo
- Falta de transporte
- Falta de recursos por el pasaje
- Falta de recursos por tratamiento
- Malos servicios de salud
- Barrera del idioma
- Demasiada gente en el hospital
- No podia dejar a los niños
- Se automedicó
- El esposo no permite/machismo
- La religion
- Otra
- No quiere contestar
- No sabe

4.12.5.2.2) Si Otra razon no hospital

4.12.5.2.2.1) Especifique

4.13) ¿Tomó medicamento para tratar esta diarrea?

- ○ Sí
- ○ No
- ○ No quiere contestar
- ○ No sabe

4.14) Si tomó medicamento para tratar esta diarrea

4.14.1) ¿Tomó antibióticos para tratar esta diarrea?

- ○ Sí
- ○ No
- ○ No quiere contestar
- ○ No sabe

4.15) Si residente es <5 años

4.15.1) ¿Tomo sueros o sobres?

- ○ Sí
- ○ No
- ○ No quiere contestar
- ○ No sabe

4.15.2) Si tomó sueros

4.15.2.1) ¿De dónde consigio estos sueros or sobres? (No lea las opciones; marque todas las que apliquen).

- ☐ Puesto de Salud
- ☐ Centro de Salud
- ☐ Hospital público
- ☐ Hospital privado
- ☐ Clínica privada
- ☐ Centro de convergencia
- ☐ Trabajador de salud

- [ ] Jornada médica
- [ ] Farmacia
- [ ] Tienda
- [ ] Medicina tradicional/natural
- [ ] Miembros de la familia
- [ ] Otro
- [ ] No quiere contestar
- [ ] No sabe

4.15.2.2) ¿Qué tipo de suero tomó?

- [ ] Suero Tomado
- [ ] Sobrecito
- [ ] No sabe

4.15.2.3) Si tomo suero en sobrecito

4.15.2.3.1) ¿Cuál clase de suero o sal oral recibió?

- [ ] A
- [ ] B
- [ ] C
- [ ] D
- [ ] E
- [ ] F
- [ ] G
- [ ] H
- [ ] I
- [ ] J
- [ ] K
- [ ] No Sabe

4.15.2.3.2) Si recibió A:

4.15.2.3.2.1) ¿Cuántos sobrecitos A utilizó durante la enfermedad?

4.15.2.3.2.2) ¿Por cuántos días utilizó los sobrecitos A?

4.15.2.3.2.3) ¿En cuánta cantidad de agua disolvió cada sobrecito A?

- Menos de 1/4 litro(<1 vaso)
- Un cuarto de litro (1 vaso)
- Medio litro (2 vasos)
- Un litro (4 vasos)
- Más de un litro (>4 vasos)
- No sabe

4.15.2.3.3) Si recibió B:

4.15.2.3.3.1) ¿Cuántos sobrecitos B utilizó durante la enfermedad?

4.15.2.3.3.2) ¿Por cuántos días utilizó los sobrecitos B?

4.15.2.3.3.3) ¿En cuánta cantidad de agua disolvió cada sobrecito B?

- Menos de 1/4 litro(<1 vaso)
- Un cuarto de litro (1 vaso)
- Medio litro (2 vasos)
- Un litro (4 vasos)
- Más de un litro (>4 vasos)
- No sabe

4.15.2.3.4) Si recibió C:

4.15.2.3.4.1) ¿Cuántos sobrecitos C utilizó durante la enfermedad?

4.15.2.3.4.2) ¿Por cuántos días utilizó los sobrecitos C?

4.15.2.3.4.3) ¿En cuánta cantidad de agua disolvió cada sobrecito C?

- Menos de 1/4 litro(<1 vaso)
- Un cuarto de litro (1 vaso)

- Medio litro (2 vasos)
- Un litro (4 vasos)
- Más de un litro (>4 vasos)
- No sabe

4.15.2.3.5) Si recibió D:

4.15.2.3.5.1) ¿Cuántos sobrecitos D utilizó durante la enfermedad?

4.15.2.3.5.2) ¿Por cuántos días utilizó los sobrecitos D?

4.15.2.3.5.3) ¿En cuánta cantidad de agua disolvió cada sobrecito D?

- Menos de 1/4 litro(<1 vaso)
- Un cuarto de litro (1 vaso)
- Medio litro (2 vasos)
- Un litro (4 vasos)
- Más de un litro (>4 vasos)
- No sabe

4.15.2.3.6) Si recibió E:

4.15.2.3.6.1) ¿Cuántos sobrecitos E utilizó durante la enfermedad?

4.15.2.3.6.2) ¿Por cuántos días utilizó los sobrecitos E?

4.15.2.3.6.3) ¿En cuánta cantidad de agua disolvió cada sobrecito E?

- Menos de 1/4 litro(<1 vaso)
- Un cuarto de litro (1 vaso)
- Medio litro (2 vasos)
- Un litro (4 vasos)
- Más de un litro (>4 vasos)
- No sabe

4.15.2.3.7) Si recibió F:

4.15.2.3.7.1) ¿Cuántos sobrecitos F utilizó durante la enfermedad?

4.15.2.3.7.2) ¿Por cuántos días utilizó los sobrecitos F?

4.15.2.3.7.3) ¿En cuánta cantidad de agua disolvió cada sobrecito F?

- Menos de 1/4 litro(<1 vaso)
- Un cuarto de litro (1 vaso)
- Medio litro (2 vasos)
- Un litro (4 vasos)
- Más de un litro (>4 vasos)
- No sabe

4.15.2.3.8) Si recibió G:

4.15.2.3.8.1) ¿Cuántos sobrecitos G utilizó durante la enfermedad?

4.15.2.3.8.2) ¿Por cuántos días utilizó los sobrecitos G?

4.15.2.3.8.3) ¿En cuánta cantidad de agua disolvió cada sobrecito G?

- Menos de 1/4 litro(<1 vaso)
- Un cuarto de litro (1 vaso)
- Medio litro (2 vasos)
- Un litro (4 vasos)
- Más de un litro (>4 vasos)
- No sabe

4.15.2.3.9) Si recibió H:

4.15.2.3.9.1) ¿Cuántos sobrecitos H utilizó durante la enfermedad?

4.15.2.3.9.2) ¿Por cuántos días utilizó los sobrecitos H?

4.15.2.3.9.3) ¿En cuánta cantidad de agua disolvió cada sobrecito H?

- Menos de 1/4 litro(<1 vaso)
- Un cuarto de litro (1 vaso)
- Medio litro (2 vasos)
- Un litro (4 vasos)
- Más de un litro (>4 vasos)
- No sabe

4.15.2.3.10) Si recibió I:

4.15.2.3.10.1) ¿Cuántos sobrecitos I utilizó durante la enfermedad?

4.15.2.3.10.2) ¿Por cuántos días utilizó los sobrecitos I?

4.15.2.3.10.3) ¿En cuánta cantidad de agua disolvió cada sobrecito I?

- Menos de 1/4 litro(<1 vaso)
- Un cuarto de litro (1 vaso)
- Medio litro (2 vasos)
- Un litro (4 vasos)
- Más de un litro (>4 vasos)
- No sabe

4.15.2.3.11) Si recibió J:

4.15.2.3.11.1) ¿Cuántos sobrecitos J utilizó durante la enfermedad?

4.15.2.3.11.2) ¿Por cuántos días utilizó los sobrecitos J?

4.15.2.3.11.3) ¿En cuánta cantidad de agua disolvió cada sobrecito J?

- Menos de 1/4 litro(<1 vaso)
- Un cuarto de litro (1 vaso)

- Medio litro (2 vasos)
- Un litro (4 vasos)
- Más de un litro (>4 vasos)
- No sabe

4.15.2.3.12) Si recibió K:

4.15.2.3.12.1) ¿Cuántos sobrecitos K utilizó durante la enfermedad?

4.15.2.3.12.2) ¿Por cuántos días utilizó los sobrecitos K?

4.15.2.3.12.3) ¿En cuánta cantidad de agua disolvió cada sobrecito K?

- Menos de 1/4 litro(<1 vaso)
- Un cuarto de litro (1 vaso)
- Medio litro (2 vasos)
- Un litro (4 vasos)
- Más de un litro (>4 vasos)
- No sabe

4.15.3) ¿Durante la enfermedad de diarrea qué cantidad de alimentos recibió su niño? Eso incluye pecho, pacha, y otros líquidos.

- Menos cantidad que lo usual
- Igual cantidad que lo usual
- Más cantidad que lo usual
- No quiere contestar
- No sabe

Hogar
Caracteristicas

1) ¿En total cuántos cuartos tiene en su hogar?

2) ¿Y cuántos cuartos (habitaciones) usan principalmente para dormir?

3) ¿Tiene en su hogar un lugar (cuarto) que usan exclusivamente para

cocinar?

- Sí
- No

4) ¿El lugar que usan para cocinar lo usan sólo los miembros de su hogar o lo comparten?

- Privado
- Compartido
- No cocinan
- No quiere contestar

5) ¿Cuál es la material principal del piso?

- Piso natural (tierra/arena)
- Ladrillo de barro o tierra
- Piso rustico(tablas de madera)
- Madera lustrada
- Ladrillo de cemento o granito
- Piso ceramico
- Torta de cemento
- Otra

6) ¿Cuál es la material principal del techo?

- Paja/pajon/palma
- Teja
- Lamina de zinc/metálica
- Lamina de asbesto (duralita)
- Concreto/loza/terraza
- Otra

7) ¿Cuál es la material principal de las paredes?

- Bajareque
- Adobe
- Block
- Lamina
- Madera

- ☐ Ladrillo de barro
- ☐ Material de desechos
- ☐ Otra

8) ¿Tiene en su casa: Luz eléctrica?

- ☐ Sí
- ☐ No
- ☐ No quiere contestar

9) Si tiene luz eléctrica

9.1) ¿Cuántas bombillas hay en su casa? (Si NS=99; si No quiere contestar=94).

[ ]

10) ¿Tiene en su casa: Televisor?

- ☐ Sí
- ☐ No
- ☐ No quiere contestar

11) ¿Tiene en su casa: Radio/grabadora?

- ☐ Sí
- ☐ No
- ☐ No quiere contestar

12) ¿Tiene en su casa: Teléfono de línea o fijo?

- ☐ Sí
- ☐ No
- ☐ No quiere contestar

13) ¿Tiene en su casa: Teléfono celular?

- ☐ Sí
- ☐ No
- ☐ No quiere contestar

14) ¿Tiene en su casa: Carro o pick-up?

- ☐ Sí
- ☐ No
- ☐ No quiere contestar

15) ¿Tiene en su casa: Moto?

- [ ] Sí
- [ ] No
- [ ] No quiere contestar

16) ¿Tiene en su casa: Bicicleta?

- [ ] Sí
- [ ] No
- [ ] No quiere contestar

17) ¿Tiene en su casa: Refrigeradora?

- [ ] Sí
- [ ] No
- [ ] No quiere contestar

18) ¿Tiene en su casa: Lavadora de ropa?

- [ ] Sí
- [ ] No
- [ ] No quiere contestar

19) ¿Tiene en su casa: Secadora de ropa?

- [ ] Sí
- [ ] No
- [ ] No quiere contestar

20) ¿Tiene en su casa: Microondas?

- [ ] Sí
- [ ] No
- [ ] No quiere contestar

21) ¿Tiene en su casa: Computadora?

- [ ] Sí
- [ ] No
- [ ] No quiere contestar
