# Supplementary material for: Incidence and etiology of infectious diarrhea from a facility-based surveillance system in Guatemala, 2008–2012
Source: BMC Public Health. 2019 Oct 22;19:1340. doi: 10.1186/s12889-019-7720-2 (PMC6805345; doi:10.1186/s12889-019-7720-2)
Supplement: Supplementary file 2 — Additional file 2. Surveillance Questions. Questions used for patients enrolled in the surveillance system. [file 12889_2019_7720_MOESM2_ESM.pdf]

CDC-CAP/UVG-CES
Programa Internacional de Enfermedades Emergentes
Proyecto de Vigilancia Comunitaria – Santa Rosa
Formulario de Admisión (H3) en el Hospital de Cuilapa
*-

UNIDAD A

1. Primeros nombres del paciente: ______________________
2. Apellidos del paciente: ______________________
3. Edad en AÑOS *(ponga 0 si tiene menos de 1 año)* ☐ ☐
   3.1. Si MENOS DE 1 AÑO, edad en MESES *(ponga 0 si tiene menos de 1 mes)* ☐ ☐
   3.2. Si MENOS DE 1 MES, edad en DIAS ☐ ☐
4. Fecha de nacimiento: ☐ ☐/☐ ☐/☐ ☐ ☐ ☐
5. Sexo: Masculino ☐ Femenino ☐
6. Parentesco del responsable del paciente con este:
   Paciente ☐ Padre/Madre ☐ Esposo/Esposa ☐ Otro pariente ☐ Guardián legal ☐
7. Nombre del responsable del paciente: ______________________
8. Doctor que está tratando al paciente: ______________________

Información sobre enfermedades crónicas y vacunaciones

9. Tiene el paciente algún historial de enfermedades crónicas tales:

9.1. Asma	Si ☐	No ☐	NS ☐
9.2. Diabetes	Si ☐	No ☐	NS ☐
9.3. Cáncer	Si ☐	No ☐	NS ☐
9.4. Enfermedad del corazón	Si ☐	No ☐	NS ☐
9.5. Enfermedad del hígado	Si ☐	No ☐	NS ☐
9.6. Enfermedad del riñón	Si ☐	No ☐	NS ☐
9.7. Enfermedad de los pulmones	Si ☐	No ☐	NS ☐
9.8. VIH/SIDA	Si ☐	No ☐	NS ☐

9.9. Otras enfermedades neurológicas Si ☐ No ☐ NS ☐

Si el paciente es mayor de 18 años:

9.10. Hipertensión Si ☐ No ☐ NS ☐ NA ☐

Si el paciente es menor de 2 años:

9.11. Nacimiento prematuro Si ☐ No ☐ NS ☐ NA ☐

Si una de las respuesta es SI, especificar en la Hoja de Información

10. Tiene consigo la ficha de vacunación? Si ☐ No ☐

Proceda aunque la ficha no este disponible.

11. Favor indicar las vacunas que el paciente ha recibido:

11.1. La triple o solo sarampión Si ☐ No ☐ NS ☐

11.1.1. ¿Cuantas dosis? 1 ☐ 2 ☐ NS ☐

11.2. Pentavalente (DPT/Hib, Hepatitis B)? Si ☐ No ☐ NS ☐

11.2.1. ¿Cuantas dosis? 1 ☐ 2 ☐ 3 ☐ 4 ☐ NS ☐

UNIDAD B

10. Fecha de admisión: ☐ ☐/☐ ☐/☐ ☐ ☐ ☐

11. Hora de admisión: ☐ ☐:☐ ☐

12. ¿Tuvo el paciente otras hospitalizaciones de más de UNA noche en el mes anterior a la admisión actual? Si ☐ No ☐ NS ☐

12.1. ¿A cuál hospital fue ingresado? ______________________________

12.2. ¿Cuántos noches duró la hospitalización más reciente? ☐ ☐

12.3. ¿Cuántos días antes de la actual admisión pasaron después que finalizara la anterior? ☐ ☐

13. ¿Ha tomado algún medicamento en las últimas 24 horas? Si ☐ No ☐ NS ☐

Si SI :

13.1. Antibióticos Si ☐ No ☐ NS ☐

13.2. Antivirales Si ☐ No ☐ NS ☐

13.3. Anti-piréticos Si ☐ No ☐ NS ☐

13.4. Analgésicos Si ☐ No ☐ NS ☐

13.5. Esteroides Si ☐ No ☐ NS ☐

13.6. Hierbas, tes, remedio casero o natural Si ☐ No ☐ NS ☐

13.7. Jarabe para la tos Si ☐ No ☐ NS ☐

13.8. Otro Si ☐ No ☐

Si otro, especifique ____

Si el niño(a) es menor de 5 años:

14. ¿Ha tomado suero o sales en los últimos **3** días? Si ☐ No ☐ NA ☐

UNIDAD C

15. Buscó el paciente tratamiento para esta enfermedad en algún lugar/persona antes de venir al Centro de Salud? Si ☐ No ☐ NS ☐

15.1. Si **SI**, a donde fue PRIMERO por tratamiento antes de venir al hospital por esta enfermedad? ____

15.2. Que tipo de establecimiento es este? (*Marque solamente una opción)*

Hospital público ☐

Hospital privado ☐

Centro de salud ☐

Clínica privada ☐

Puesto de salud ☐

Centro de convergencia ☐

Farmacia ☐

Tienda ☐

Otra ☐

15.3. Que tratamiento(s) recibió durante esta visita?

15.3.1. Antibióticos Si ☐ No ☐ NS ☐

15.3.2. Antidiarreicos Si ☐ No ☐ NS ☐

15.3.3. Sueros o sales caseros para hidratación Si ☐ No ☐ NS ☐

15.3.4. Sueros o sales para hidratación comprados en la tienda Si ☐ No ☐ NS ☐

15.3.5. Hierbas, tes, remedios naturales Si ☐ No ☐ NS ☐

16. Buscó el paciente tratamiento para esta enfermedad en un SEGUNDO lugar/persona antes de venir al Centro de Salud? Si ☐ No ☐ NS ☐

16.1. Cual fue el SEGUNDO lugar a donde acudió en busca de tratamiento? __________

16.2. Que tipo de establecimiento es este? (*Marque solamente una opción)*

Hospital público ☐

Hospital privado ☐

Centro de salud ☐

Clínica privada ☐

Puesto de salud ☐

Centro de convergencia ☐

Farmacia ☐

Tienda ☐

Otra ☐

16.3. Que tratamiento(s) recibió durante esta visita?

16.3.1. Antibióticos Si ☐ No ☐ NS ☐

16.3.2. Antidiarreicos Si ☐ No ☐ NS ☐

16.3.3. Sueros o sales caseros para hidratación Si ☐ No ☐ NS ☐

16.3.4. Sueros o sales para hidratación comprados en la tienda Si ☐ No ☐ NS ☐

16.3.5. Hierbas, tes, remedios naturales Si ☐ No ☐ NS ☐

17. Buscó el paciente tratamiento para esta enfermedad en un TERCER lugar/persona antes de venir al Centro de Salud? Si ☐ No ☐ NS ☐

17.1. Cual fue el TERCER lugar a donde acudió en busca de tratamiento? ____________

17.2. Que tipo de establecimiento es este? (*Marque solamente una opción)*

Hospital público ☐

Hospital privado ☐

Centro de salud ☐

Clínica privada ☐

Puesto de salud ☐

Centro de convergencia ☐

Farmacia ☐

Tienda ☐

Otra ☐

17.3. Que tratamiento(s) recibió durante esta visita?

17.3.1. Antibióticos Si ☐ No ☐ NS ☐

17.3.2. Antidiarreicos Si ☐ No ☐ NS ☐

17.3.3. Sueros o sales caseros para hidratación Si ☐ No ☐ NS ☐

17.3.4. Sueros o sales para hidratación comprados en la tienda Si ☐ No ☐ NS ☐

17.3.5. Hierbas, tes, remedios naturales Si ☐ No ☐ NS ☐

Ahora quisiera hacerle unas preguntas sobre su episodio de diarrea.

18. Actualmente tiene diarrea? Si ☐ No ☐

18.1. Si no, hace cuántos días la tuvo? ☐ ☐

19. Diarrea con sangre Si ☐ No ☐ NS ☐

20. Diarrea con moco Si ☐ No ☐ NS ☐

21. Náusea con vómitos Si ☐ No ☐ NS ☐

21.1. Si **SI**, número de días en la semana pasada en los que tuvo ☐ ☐

21.2. Actualmente tiene náusea con vómitos? Si ☐ No ☐

22. Calambres o dolor abdominal Si ☐ No ☐ NS ☐

23. Letargia (con tendencia al sueño) Si ☐ No ☐ NS ☐

24. Tiene el paciente alguna condición intestinal como Colitis ulcerativa, Enfermedad inflamatoria gastrointestinal, Enfermedad de Crohn, Enfermedad celiaca, Intolerancia lactosa u otros síndromes de mala absorción? Si ☐ No ☐ NS ☐

Para niños < 5 años pregúntale las siguientes:

25. Bebe ávidamente con sed mayor de lo usual Si ☐ No ☐ NS ☐

26. Bebe muy mal o no puede beber Si ☐ No ☐ NS ☐

27. Irritable/incómodo/intranquilo Si ☐ No ☐ NS ☐

****

28. Inscrito como caso de enfermedad respiratoria? Si ☐ No ☐

****

UNIT 2.2 "D"

Entrevista para casos respiratorios

29. Fiebre Si ☐ No ☐ NS ☐

29.1. ¿Cuándo comenzó? ☐☐/☐☐/☐☐☐☐

30. Escalofríos Si ☐ No ☐ NS ☐

30.1. ¿Cuándo comenzó? ☐☐/☐☐/☐☐☐☐

31. Tos Si ☐ No ☐ NS ☐

31.1. ¿Cuándo comenzó? ☐☐/☐☐/☐☐☐☐

32. Produce esputo Si ☐ No ☐ NS ☐

32.1. ¿Cuándo comenzó? ☐☐/☐☐/☐☐☐☐

33. Esputo con sangre Si ☐ No ☐ NS ☐

33.1. ¿Cuándo comenzó? ☐☐/☐☐/☐☐☐☐

34. Dificultad para respirar Si ☐ No ☐ NS ☐

34.1. ¿Cuándo comenzó? ☐☐/☐☐/☐☐☐☐

35. Dolor de pecho al respirar Si ☐ No ☐ NS ☐

35.1. ¿Cuándo comenzó? ☐☐/☐☐/☐☐☐☐

36. Dolor de garganta Si ☐ No ☐ NS ☐

36.1. ¿Cuándo comenzó? ☐☐/☐☐/☐☐☐☐

37. Le gotea la nariz Si ☐ No ☐ NS ☐

38. Estornudos Si ☐ No ☐ NS ☐

39. Ruidos al respirar o falta de aire Si ☐ No ☐ NS ☐

40. Dolor de cabeza Si ☐ No ☐ NS ☐

41. Dolor muscular Si ☐ No ☐ NS ☐

42. Vómitos Si ☐ No ☐ NS ☐

43. Diarrea Si ☐ No ☐ NS ☐

****

44. Inscrito como caso con fiebre de origen no-específico? Si ☐ No ☐

Si, favor pasar a la Unit 2.3 "E" Encuesta de enfermedad febril, Pregunta 47.

Si NO, favor pasar a la Unit 2.4 "F", Encuesta de factores de riesgo, Pregunta 48.

UNIT 2.3 "E"

Interview questions for fever of unknown origin patients

45. Ha tenido el paciente alguno de los siguientes síntomas en los últimos 3 días?

	3d	Hoy	No Sabe		Si SI, fecha de inicio
57. Dolor de cabeza	☐	☐	☐	☐	☐☐/☐☐/☐☐☐☐
58. Dolor retroorbital	☐	☐	☐	☐	☐☐/☐☐/☐☐☐☐
59. Sensibilidad liviana	☐	☐	☐	☐	☐☐/☐☐/☐☐☐☐
60.Ojos rojos	☐	☐	☐	☐	☐☐/☐☐/☐☐☐☐
61. Ojos amarillos	☐	☐	☐	☐	☐☐/☐☐/☐☐☐☐

62. Escalofríos □ □ □ □ □□/□□/□□□□

63. Sudoración profusa □ □ □ □ □□/□□/□□□□

64. Orina obscura □ □ □ □ □□/□□/□□□□

65. Mialgias □ □ □ □

□□/□□/□□□□

66.Dolor en los camotes □ □ □ □ □□/□□/□□□□

67.Artralgias □ □ □ □ □□/□□/□□□□

68.Rigidez en la nuca □ □ □ □ □□/□□/□□□□

69.Debilidad facial/muscular □ □ □ □ □□/□□/□□□□

70.Dormido el cuerpo □ □ □ □ □□/□□/□□□□

71.Dificultad para caminar □ □ □ □ □□/□□/□□□□

72.Vómitos □ □ □ □ □□/□□/□□□□

73.Vómitos con sangre □ □ □ □ □□/□□/□□□□

74.Dolor abdominal □ □ □ □ □□/□□/□□□□

75.Diarrea □ □ □ □ □□/□□/□□□□

76.Náusea □ □ □ □ □□/□□/□□□□

77.Irritación en la piel □ □ □ □ □□/□□/□□□□

77.1 Si **SI**, dónde? Cara □ Tórax □ Brazos/manos □ Piernas/pies

77.2 Tipo de irritación:

Macular □ Papular □ Vesicular □ Pustular □ Otra □

77.3 Se tomó foto de la irritación? Si □ No □

78.Costras? □ □ □ □ □□/□□/□□□□

79.Sangrado inusual □ □ □ □ □□/□□/□□□□

79.1 Si SI, Encías ☐ Nariz ☐ Heces/obscuras/alquitranadas ☐ Fácil para moretes ☐ Otros ☐

UNIT 2.4 “F”

Entrevista de factores de riesgo

Factores de riesgo

46. Cuántos dormitorios hay en su casa? ☐ ☐

47. Cuántas personas viven en su casa? ☐ ☐

48. Grupo étnico:

48.1. Indígena? ☐

48.2. Ladino? ☐

48.3. Garífuna? ☐

48.4. Xinca? ☐

48.5. Otro? ☐

49. Cuál fue el último grado escolar que el paciente (guardian if patient is <18) completó?

Ninguno ☐ Primaria ☐ Básico ☐ Secundaria ☐ Estudios superiores ☐

50. Ingresos mensuales de la familia:

< Q1000 ☐ Q1001-Q3000 ☐ Q3001-Q5000 ☐ > Q5000 ☐ No responde ☐

51. De que material esta hecho el techo de su casa? Teja ☐ Palma ☐ lamina ☐ otro ☐

52. Qué tipo de piso tienen en su casa? Tierra ☐ Cemento ☐ Piedra/otro ☐

53. Tienen cedazo en las ventanas de su casa? Si ☐ No ☐

54. Tienen en su casa alguno de los siguientes (*marque todos los que apliquen*)?

54.1. Energía eléctrica Si ☐ No ☐

54.1.1. ¿Cuántas bombillas hay en su casa al día de hoy? _____

54.2. Refrigeradora Si ☐ No ☐

54.3. Computadora Si ☐ No ☐

54.4. Radio Si ☐ No ☐

54.5. Lavadora Si ☐ No ☐

54.6. Carro/Camión Si ☐ No ☐

54.7. Televisión Si ☐ No ☐

54.8. Secadora Si ☐ No ☐

54.9. Teléfono Si ☐ No ☐

54.10. Microondas Si ☐ No ☐

54.11. De dónde sacan agua para beber o cocinar? *(No lea las opciones, seleccione todas las que apliquen)*

- Chorro dentro de la casa de la red pública ☐
- Chorro en el patio/compartido de red pública (chorro público) ☐
- Chorro en el patio/compartido con otra fuente ☐
- Lavaderos públicos ☐
- Tienen pozo propio ☐
- Pozo público ☐
- Compran agua pura /agua embotellada ☐
- Agua de camión o cisterna ☐
- Agua de lluvia ☐
- Agua del río o lago ☐

55. Ustedes normalmente almacenan agua para beber en su casa? Si ☐ No ☐

55.1. Generalmente le hacen algo al agua para limpiarla [o tratan el agua] antes de beberla? Si ☐ No ☐

55.1.1. La hierven Si ☐ No ☐

55.1.2. Le agregan químicos (cloro) Si ☐ No ☐

55.1.3. La filtran (con tela o filtro cerámico) Si ☐ No ☐

55.1.4. ¿Qué más le hacen? ____________________

56. Usan repelentes contra los mosquitos? Si ☐ No ☐

57. Usan pabellón o mosquiteros para dormir? Si ☐ No ☐

58. Usted fuma? Si ☐ No ☐

59. Alguien en su casa fuma? Si ☐ No ☐

60. Manda a su niño a una guardería infantil? Si ☐ No ☐ NA ☐

61. Si el paciente es menor de un (1) año: ¿Al niño le dieron de pecho durante el primer año de vida? Si ☐ No ☐

61.1. ¿Durante los 7 días antes de que el niño se enfermara, que comió el niño?

61.1.1. Solamente formula infantil Si ☐ No ☐

61.1.2. Solamente pecho Si ☐ No ☐

61.1.3. Formula infantil y pecho Si ☐ No ☐

61.1.4. Ni formula infantil, ni pecho Si ☐ No ☐

62. ¿En el mes antes de enfermarse ha viajado fuera de Guatemala? Si ☐ No ☐

63. ¿En el mes antes de enfermarse visitó otro departamento ? Si ☐ No ☐

64. ¿En el mes antes de enfermarse lo mordió algún animal? Si ☐ No ☐

65. ¿Lo picó alguna garrapata en las 2 semanas antes de enfermarse? Si ☐ No ☐

66. ¿Lo picó alguna pulga en las 2 semanas antes de enfermarse? Si ☐ No ☐

67. Hay ahora alguna otra persona en su casa que esté enferma con fiebre, dolor de garganta o tos? Si ☐ No ☐

68. ¿A usted / al paciente le ha dado dengue alguna vez? Si ☐ No ☐

   68.1. Cuando (Año): ☐ ☐ ☐ ☐

   68.2. Cuando (Mes): ☐ ☐

   68.3. Se lo diagnosticaron algún personal médico? Si ☐ No ☐

69. Ha visto ratones u otros roedores en su casa durante el mes anterior? Si ☐ No ☐

70. En los últimos días, ha usted tocado o ha estado a un metro de distancia de cualquiera de los siguientes pájaros o animales? (*Marque todos los que apliquen*)

- Pollos o gallinas ☐
- Patos/Gansos ☐
- Cerdos ☐
- Palomas ☐
- Gallos de pelea ☐
- Otros pájaros ☐

   70.1. Si contesta que **SI** a cualquier pájaro o animal en la pregunta 15, estaba cualquiera de estos pájaros o animales enfermos o muertos? Si ☐ No ☐ NS ☐

   70.1.1. Indicar cuál de los animales estaba enfermo o muerto.

- Pollos / gallinas ☐
- Patos / Gansos ☐

Cerdos ☐

Palomas ☐

Gallos de pelea ☐

Otros pájaros ☐

Si cualquiera de las preguntas arriba es SI, favor de especificarlo en la Hoja de Información

****

If Diarrhea Patient, go to Unit 3.1 “G”, Patient Exam, Question 74

If Respiratory Patient go to Unit 3.2 “H”, Patient Exam, Question.

****

UNIT 3.1 “G”

Diarrea Examen Fisico hecho por la enfermera

71. Verifique los siguientes signos para ninos < *5 anos* :

71.1. Ojos:

Normal ☐ Hundidos (madre confirma que mas de lo normal) ☐

71.2. Mucosa oral:

Normal ☐ Algo sec ☐ Bastante seca ☐

71.3. Sed:

Normal ☐ Con sed, bebe ávidamente ☐ Toma poco o nada ☐

71.4. Pellizco de piel:

Normal ☐ Demora poco (≤2 sec.) ☐ Demora mucho (>2 sec.) ☐

71.5. Mollera hundida:

Normal ☐ Hundidos (madre confirma que mas de lo normal) ☐

71.6. Estado mental:

Normal ☐ inquieto, irritable ☐ Letárgico/inconsciente ☐

72. Temperatura (°C) al presentarse ☐ ☐ . ☐ °C

****

If Enrolled as Diarrhea, go to Unit 4.1 “J”, Chart Review, Question85.

If Enrolled as Respiratory, go to Unit 3.2 “H” , Patient Exam, Question 73 .

****

UNIT 3.2"H"

Examen medico para pacientes con enfermedad respiratoria

73. Auscultación:

73.1.	Sibilancias	Si ☐	No ☐	NA ☐
73.2.	Estertores gruesos	Si ☐	No ☐	NA ☐
73.3.	Estertores finos	Si ☐	No ☐	NA ☐
73.4.	Roncus	Si ☐	No ☐	NA ☐

74. Adenopatia	Si ☐	No ☐	NA ☐
75. Estado mental alterado	Si ☐	No ☐	NA ☐

Para niños <12 años:

76. Aleteo nasal	ninguno ☐	intermitente ☐	siempre ☐
77. Murmullo vesicular	normal ☐	sonido leve ☐	ausente ☐
78. Usa músculos del pecho para respirar:	leve ☐	moderado ☐	grave ☐
79. Puntuación de Dawnes	0-3 ☐	4-5 ☐	>6 ☐

Para niños <2 años:

80. Letárgico	Si ☐	No ☐	NA ☐
81. No puede comer o beber bien	Si ☐	No ☐	NA ☐

****

If the patient is enrolled in respiratory diseases g go to unit 4.2 "K", question 87.

UNIT 4.1 “J”

Record medico para casos de diarreas

82. Diagnóstico:

82.1.	Neumonía	Si ☐	No ☐
82.2.	Infección del tracto respiratorio superior (IRS)	Si ☐	No ☐
82.3.	Sonidos como de pito/broncoespasmo	Si ☐	No ☐
82.4.	Enfermedades crónico-destructivas pulmonares (EPOC)	Si ☐	No ☐
82.5.	Influenza o enfermedad similar a influenza	Si ☐	No ☐
82.6.	Faringitis/amigdalitis	Si ☐	No ☐
82.7.	Otitis media	Si ☐	No ☐
82.8.	Diarrea	Si ☐	No ☐
82.9.	Gastroenteritis	Si ☐	No ☐
82.10.	Desintería	Si ☐	No ☐
82.11.	Deshidración	Si ☐	No ☐
82.12.	Otro	Si ☐	No ☐

82.12.1. Especificar? ____________________

****

If Enrolled as Diarrhea, go to Unit 5, Taking Specimens, Question 94.

If Enrolled as Respiratory, go to Unit 4.2 “K”, Chart Review, Question 87.

****

UNIT 4.2 “K”

Record medico enfermedades respiratorias

83. Presión sanguínea ☐ ☐ ☐/☐ ☐ ☐

84. Pulso por minuto ☐ ☐ ☐

85. Gas arterial

85.1.1. pH ☐ ☐.☐

85.1.2. pO_2 ☐ ☐.☐ %

85.1.3. pCO_2 ☐ ☐.☐ %

85.1.4. Saturación de oxigeno ☐ ☐.☐ %

86. Diagnósticos de admisión (*Marque todos los que apliquen*):

- Neumonía (NM) ☐
- Bronconeumonía (BNM) ☐
- Bronquitis hiperactiva ☐
- Bronquiolitis ☐
- Inyección respiratoria superior (IRS) ☐
- Dificultad respiratoria ☐

Disnea ☐

Bronquioespasmos ☐

Bronchiectasis ☐

Traquebronquitis o traquebronquiolitis ☐

Enfermedad pulmonar obstructive cronica (EPOC) ☐

Faringitis, amigdalitis, o laringotraqueitis ☐

Otitis media ☐

Diarrea ☐

Gastroenteritis ☐

Dysentery ☐

Dehydration ☐

Sepsis ☐

Asma (Estadio asmático, asma bronquial, crisis asmático) ☐

Cianosis ☐

Croup ☐

Pleuritis (o derrame pleural) ☐

Tuberculosis (TB) ☐

Tos (o tos hemoptisis) ☐

Absceso pulmonar ☐

Fiebre tifoidea ☐

Tos en excesos ☐

Fiebre ☐

Tos ferina (coquelachoina) ☐

Fatiga ☐

Dolor toráxico ☐

Otro ☐
Especifique ____________________

Tratamiento de antibióticos al momento de la admisión

87. Ha recibido el paciente alguno de estos tratamientos durante las primeras 24 horas de admisión?

87.1. Antivirales Si ☐ No ☐ NS ☐

87.1.1. ¿Cual antiviral recibió?

Amantadina Si ☐ No ☐ NS ☐

Oseltamivir Si ☐ No ☐ NS ☐

87.2. Esteroide Si ☐ No ☐ NS ☐

87.2.1. ¿Cual esteroide recibió?

Dexametazona Si ☐ No ☐ NS ☐

Hidrocortizona succinatio Si ☐ No ☐ NS ☐

Metilprednisolona Si ☐ No ☐ NS ☐

Prednisona Si ☐ No ☐ NS ☐

87.3. Suelo o sales de hidratación oral Si ☐ No ☐ NS ☐

87.4. Antibióticos Si ☐ No ☐ NS ☐

87.4.1. Marque todos los antibioticos que recibio:

Amikacina ☐

Amoxicilina ☐

Amoxicilina+ Acido clavulanico ☐

Ampicilina ☐

Ampicilina+Sulbactam ☐

Azitromicina ☐

Cefotaxima ☐

Cefotriaxone ☐

Cefalotina ☐

Cefuroxima ☐

Ciprofloxacina ☐

Clindamicina ☐

Cloranfenicol ☐

Dicloxicina ☐

Doxicilina ☐

Gentamicina ☐

Meropenem ☐

Metronidazol ☐

Ofloxacina ☐

Penicilina ☐

Trimetroprim-sulfametoxazol (TMP-SMZ) ☐

Vancomicina ☐

Otro? ☐

Especifique: ____

UNIT 5 “M”

Pruebas biológicas

88. Sangre para cultivo Fecha: ☐ ☐/☐ ☐/☐ ☐ ☐ ☐ No. de ml ☐ ☐.☐

89. Sangre para suero Fecha: ☐ ☐/☐ ☐/☐ ☐ ☐ ☐ No.de ml ☐ ☐.☐

90. Ha recibido el paciente algún antibiótico en las últimas 72 horas antes de colectar la muestra de sangre? Si ☐ No ☐ No sabe ☐

91. Heces Muestra proporcionada ☐ No seleccionada ☐ Frote usado ☐

91.1. Fecha de collecion para heces: ☐ ☐/☐ ☐/☐ ☐ ☐ ☐

91.2. ¿Muestra adecuada? Si. ☐ No. ☐

92. Ha recibido el paciente algún antibiótico en las últimas 72 horas antes de colectar la muestra de heces? Si. ☐ No. ☐

93. Frote NP Fecha:

☐ ☐/☐ ☐/☐ ☐ ☐ ☐

94. Frote OP Fecha: ☐ ☐/☐ ☐/☐ ☐ ☐ ☐

95. Orina Fecha: ☐ ☐/☐ ☐/☐ ☐ ☐ ☐ No. de ml ☐ ☐ ☐

96. .Radiografia de pecho Si ☐ No ☐

97. Otras pruebas adicionales al momento de la admisión:

Fórmula diferencial Química de la sangre ☐

Pruebas de funcionamiento del hígado ☐

Otras ☐

Especifique ______________________

Si cualquiera de las respuestas arriba es SI, favor especificarlo en la Hoja de Información

Solo para pacientes de resperiatoria:

98. Fecha para la próxima visita de seguimiento: ☐ ☐/☐ ☐/☐ ☐ ☐ ☐
